# The Biology, Microclimate, and Geology of a Distinctive Ecosystem Within the Sandstone of Hyper‐Arid Timna Valley, Israel

**DOI:** 10.1111/1758-2229.70188

**Published:** 2025-09-15

**Authors:** Irit Nir, Rachel Armoza‐Zvuloni, Hana Barak, Asunción De los Ríos, Christopher P. McKay, Ariel Kushmaro

**Affiliations:** ^1^ Avram and Stella Goldstein‐Goren Department of Biotechnology Engineering Ben‐Gurion University of the Negev Beer Sheva Israel; ^2^ Dead Sea and Arava Science Center Yotvata Israel; ^3^ Campus Eilat, Ben Gurion University Eilat Israel; ^4^ Department of Civil and Environmental Engineering Ben‐Gurion University of the Negev Beer Sheva Israel; ^5^ Department of Biogeochemistry and Microbial Ecology National Museum of Natural Sciences (MNCN‐CSIC) Madrid Spain; ^6^ Space Science Division NASA Ames Research Center Moffett Field California USA; ^7^ School of Sustainability and Climate Change Ben‐Gurion University of the Negev Beer Sheva Israel; ^8^ The Ilse Katz Center for Nanoscale Science and Technology Ben‐Gurion University of the Negev Beer Sheva Israel

**Keywords:** *Chroococcidiopsis*, hyper‐arid, litho‐bionts, microclimate, sandstone, Timna Valley

## Abstract

Microbial endolithic communities in the sandstone rocks of the southern Negev Desert, particularly in Timna Park, were initially discovered by Imre Friedmann and Roseli Ocampo‐Friedmann in their pioneering study about 50 years ago. Nonetheless, this harsh microecosystem, dominated by cyanobacterial taxa, raises questions about the adaptive mechanisms that enable the survival of these microorganisms. The present study provides comprehensive data, including extensive precipitation records for the Timna Valley, and multi‐year microclimatic data from a colonised site. It includes examinations of rock structure, as well as microscopic and metagenomic analysis. Our findings point to a distinct bacterial endolithic population dominated by the cyanobacterial genus *Chroococcidiopsis*. Although the taxa are well known, we show here how their exclusive persistence is driven by the sandstone's fine porosity and thermal properties, combined with rare, low‐volume precipitation. This highly selective microenvironment highlights how specific rock and climate interactions can filter microbial diversity in hyper‐arid deserts. Additionally, it demonstrates an adaptation strategy based on both short‐term and decadal‐scale dormancy. Thus, it offers new insights for the survival of these unique ecosystems and provides valuable perspectives for astrobiology and the search for evidence of microbial life on Mars.

## Graphical abstract

This study describes an endolithic microbial community primarily composed of the cyanobacterial taxon *Chroococcidiopsis*, along with comprehensive data regarding the local geological and microclimatic factors that affect this distinct ecosystem. Metagenomic analysis identified genes associated with adaptive mechanisms for extreme aridity, with sporadic rainfall as the primary water source.

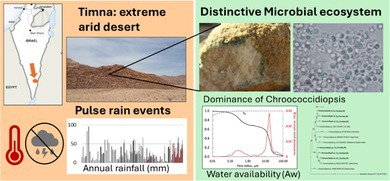

## 1 Introduction

Arid and hyper‐arid regions comprise the largest terrestrial ecosystems, covering approximately 30% of the world's land areas. Climatically, they are defined as biomes with a ratio of mean annual rainfall to mean annual evaporation of less than 0.05 for arid zones and below 0.002 for extreme hyper‐arid areas (Mohammadipanah and Wink 2016; Bull 2011). Therefore, water is a limited resource for life in hyper‐arid deserts. Constrained water areas are often accompanied by high temperatures, low nutrient concentrations, and intense radiation conditions (Bull 2011). Life in these areas is challenging, and only highly adapted forms of life can survive. One such example is microbial communities living in lithic cryptic habitats, which are well‐documented (Friedmann et al. 1988; Wierzchos et al. 2012; De los Ríos et al. 2014; De los Rios et al. 2022). However, despite the existing knowledge about specialised forms of life, questions remain regarding their distribution, survival capacity, and specific adaptations to harsh living environments. In their pioneering work in the southern Negev Desert, Israel (e.g., Timna Park), Imre Friedmann and Roseli Ocampo‐Friedmann were the first to describe microbial communities living within sandstone rocks in this desert (Friedmann et al. 1967). They reported that these lithic microbial communities were primarily composed of photosynthetic microorganisms, predominantly cyanobacteria, particularly *Chroococcidiopsis* sp., accompanied by various taxa of heterotrophic bacteria. Friedmann et al. (1967) concluded that these endolithic cyanobacteria “may represent those photoautotrophic organisms which live under the most extreme ecological conditions on the earth's surface.” (Friedmann et al. 1967; Friedmann and Ocampo‐Friedmann 1984). Interest in endolithic cyanobacteria at Timna increased following the report of similarities to endolithic cyanobacteria found in the Beacon sandstone of the Antarctic Dry Valleys (Friedmann and Ocampo 1976). However, further detailed analysis and classification of the microbial components in the endolithic communities of the Dry Valley conducted by Friedmann et al. (1988) revealed the existence of two types of eukaryotic communities (lichen‐dominated and *Hemichloris* communities) and three cyanobacteria‐dominated communities (*Gloeocapsa*, *Hormathonema‐Gloeocapsa*, and *Chroococcidiopsis*). Friedmann and Ocampo‐Friedmann (1984) concluded that in hot deserts, endolithic microbial communities consist solely of prokaryotes. The number of taxa is significantly lower compared to those in the Antarctic desert and is composed only of prokaryotes. The number of taxa is considerably lower compared to that of the Antarctic desert. They suggested that in hot deserts such as the Timna area, “A favorable combination of available water and moderate temperature range occurs in the early morning after dew fall” (Friedmann and Ocampo‐Friedmann 1984). As the sun warms the surface, the combination of a temperature increase in a hydrated state and the concomitant loss of water imposes severe environmental stress that eukaryotic organisms appear to be unable to tolerate. Based on this, they concluded that “the endolithic environment in hot deserts is much harsher and more extreme than its counterpart in the Antarctic polar desert.” (Friedmann and Ocampo‐Friedmann 1984). Years later, Qu et al. (2020), in a study of a set of sandstone endoliths that included Timna and Antarctic dry valleys, concluded that the microclimate was the primary driver of endolithic community composition. Friedmann and Ocampo‐Friedmann (1984) attributed this to the specialisation of prokaryotic and eukaryotic primary producers to different climate conditions. It is interesting to note that a similar conclusion was reached by Kidron and Büdel (2014), who determined that in the Negev Desert, both epilithic and hypolith cyanobacteria are confined to habitats supplied by rain, whereas lichens can thrive if dew is plentiful. Kidron and Büdel (2014) suggested that colonisation by cyanobacteria can serve as a bioindicator for dewless habitats in the dewy areas of the Negev Desert. Consistent with this, McKay (2016) reported hypolith cyanobacteria but no lichens at an arid site with rain but negligible dew in the northern part of the Arava Valley in Negev. De los Rios et al. (2022) found similar results in the Namib Desert, where they found both hypolithic lichen and cyanobacteria communities thriving in coastal foggy areas, whereas inland areas with a lack of fog and dew events only support the hypolithic community of cyanobacteria. The Timna lithobiontic community has been used as a model system to better understand the adaptation mechanisms of cyanobacteria to desert environmental conditions, with several studies conducted in recent decades. Cultures of cyanobacteria from Timna have been used as model desert organisms in studies investigating nitrogen starvation (Billi and Caiola 1996), and the effects of water stress (Potts et al. 1983; Scherer and Potts 1989). Although its natural habitat is nearly always dry, experiments with *Chroococcidiopsis* sp. isolated from this locality have shown that this cyanobacterium incorporates CO_2_ only when matric water potentials are above −10 MPa (Potts and Friedman 1981). This indicates a sharp drop in photosynthetic rates with decreasing water potential below 3400 kPa (*a*_*w*_ = 0.976). In their study aiming to determine the lower limit of water activity, Palmer and Friedmann (1990) found that the crypto endolithic cyanobacteria from Timna had maximum incorporation at 100% RH, but it decreased rapidly (by a factor of 7; *p* < 0.05) at 98% RH (water potential of −2.7 MPa) and was at trace levels at 95% RH (water potential of −6.9 MPa). This fact raises questions regarding the survival and adaptation mechanisms of cyanobacteria facing periods of water shortages. Moreover, this raises questions about the role of rock substrate (e.g., sandstone) in retaining moisture, thereby shaping the microbial community structure.

In this study, we focused on cyanobacteria‐dominated microbial communities inhabiting sandstone rocks in Timna Valley, Israel, following Imre Friedman's study (Friedmann 1980). Additionally, we examined a colonised sandstone sample from Timna that was stored for more than 25 years under dark and dry conditions to reveal the preservation abilities of the microbial community and structure. We believe that the colonised sandstone rock in Timna is an interesting system for studying life in extreme environments, particularly those dominated by the phylum Cyanobacteria. This study provides context for understanding the environmental challenges that shape the microbial community with an overview of the geological and climatological setting of the Timna site. Subsequently, new data on the daily and long‐term rainfall records for Timna and Arava Valley, detailed multi‐year microclimatic data from a colonised site, rock mineralogy, and rock pore structure at this site are presented. Finally, the community structure and geomicrobiology of these endolithic communities, as well as their genetic survival and resistance potential, were reviewed. The study demonstrates that the interaction between the physical properties of the local sandstone and Timna's infrequent, low‐intensity rainfall events creates selective environmental conditions that support the persistence of cyanobacteria‐dominated communities. These characteristics act as ecological filters, enabling cyanobacteria to outcompete lichens through an adaptive strategy that includes both short‐term and decadal dormancy. Thus, Timna's unique ecosystem provides a valuable context for examining the extended pulse‐reserve paradigm (EPRP), formulated for microbial communities in harsh environments, and provides important insights for the search for evidence of life on Mars.

### 1.1 Timna Park's Geological and Climatological Setting

**FIGURE 1:**
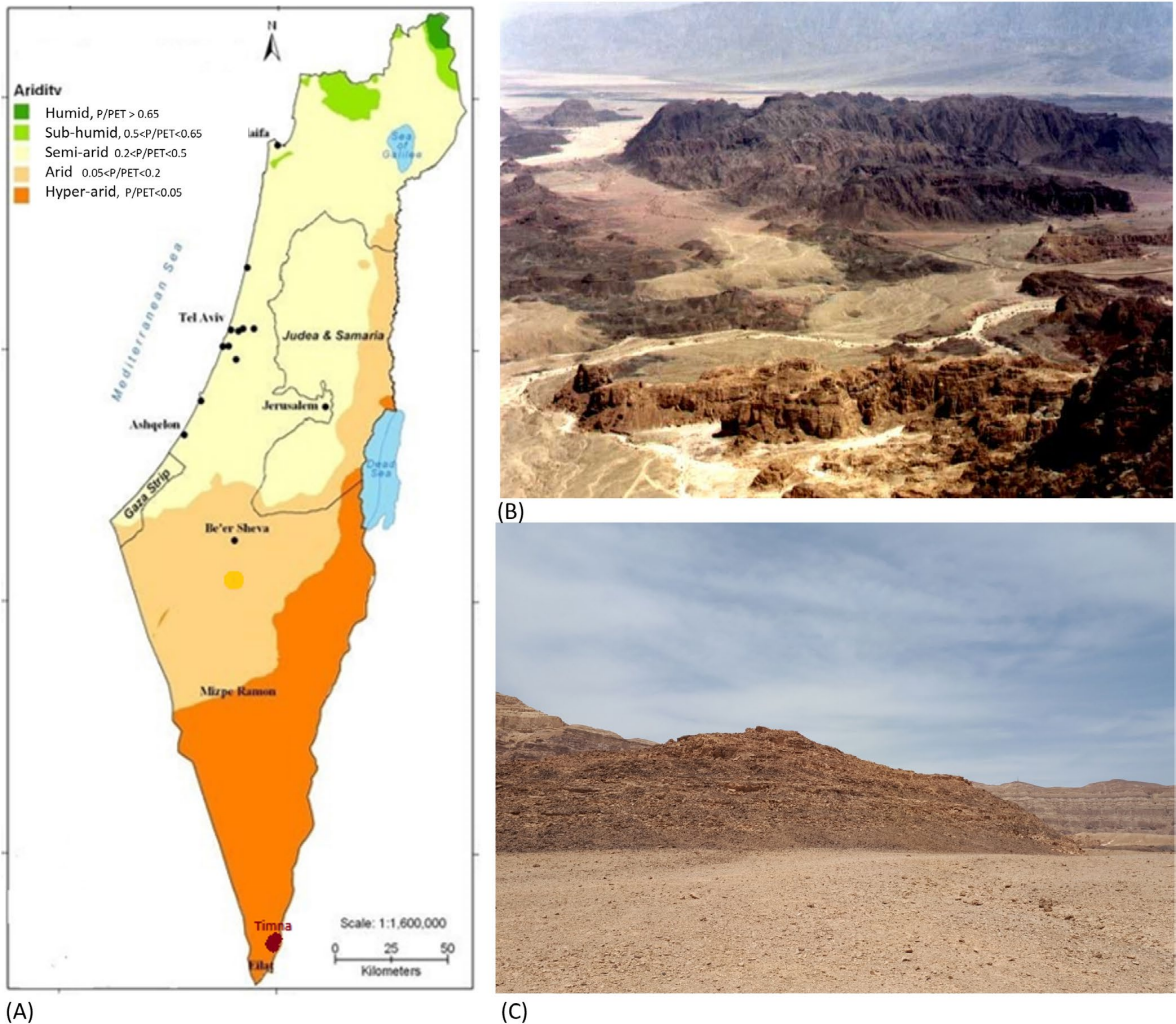
(A) Aridity index (AI) map of the southern part of Israel. Timna Park's location is highlighted in the southern part of the Arava Valley. (B) Overview of Timna's Park formations. (C) The study site location is at the top of the sandstone hill (GPS N 29° 46.225′, E 34° 56.683′, elevation: 294 m).

**FIGURE 2:**
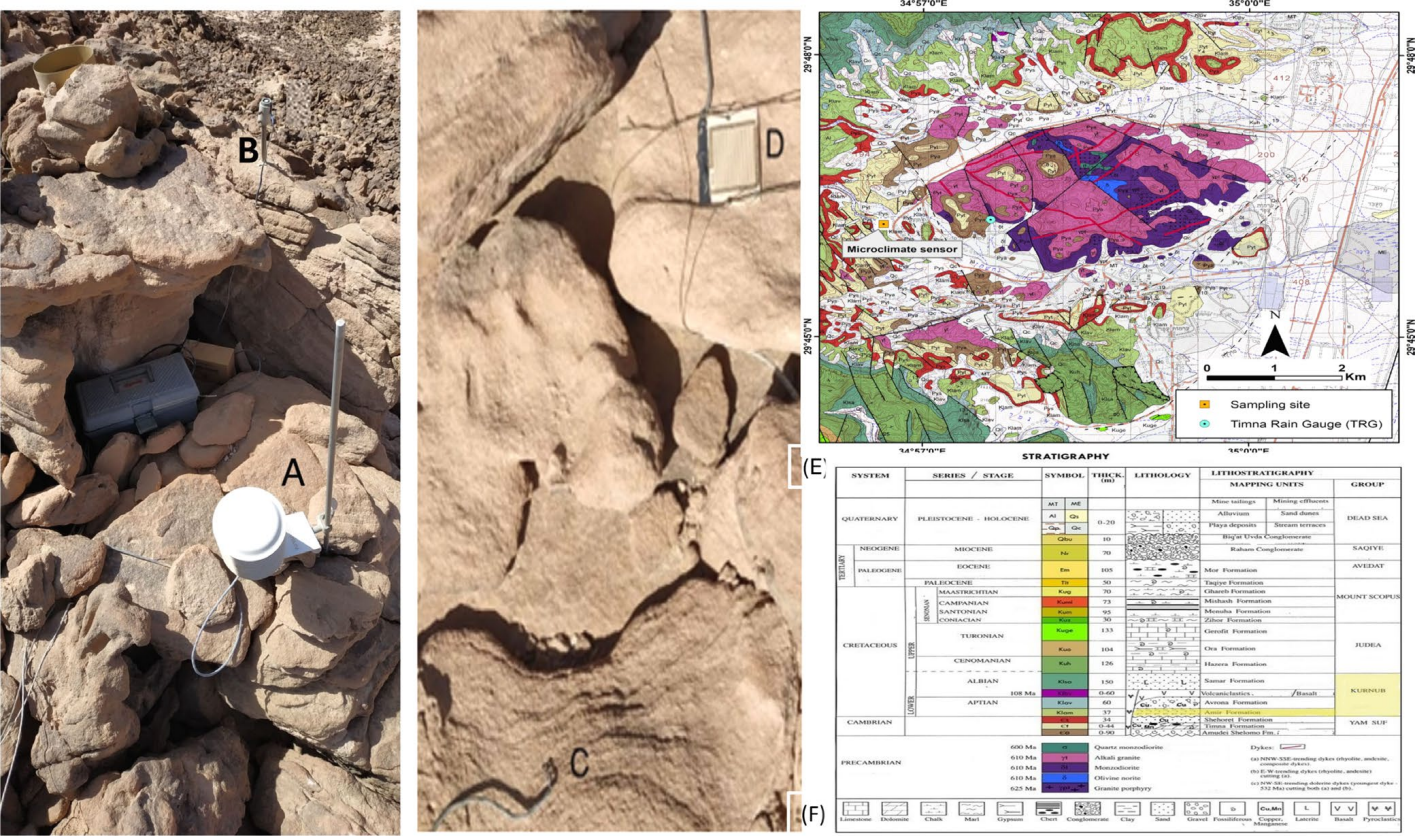
Photographs of the microclimate monitoring systems that were implemented in the study: (A) Air temperature and humidity sensor in a 12 cm diameter radiation shield. (B) Light sensor mounted ~10 cm above the rock surface in a stand. (C) Conductivity sensor (one of six). (D) Dew sensor (7 cm on a side) 4. Not shown are the rock temperature sensors and the tipping bucket rain gauge. (Photographs by Irit Nir). (E) Geological Map of Timna Park showing the location of the rain gauge in Timna Park. (F) Stratigraphy Index of the Geological map (Origin of the map and the index: Beyth et al. 1999).

Timna Park is located 25 km north of Eilat, in the Arava Valley of southern Israel, within the driest region of the Negev Desert (Figure 1A). The park is home to the world's earliest copper mining site, dating back to ancient Egyptian imperial times. Timna Park was established in 1981 by the Keren‐Kayemeth Le‐Israel Jewish National Fund (KKL‐JNF) in collaboration with the regional council and the Ministry of Tourism. The geology of Timna Park is diverse and features a range of lithologies, including plutonic rocks (granite and gabbro), marine sedimentary rocks (limestone and dolomite), sandstone formations containing copper ore, ancient alluvial terraces, and recent alluvial deposits (Beyth et al. 2018) (Figure 1B). The sandstone hill investigated in this study is located at GPS coordinates N 29° 46.225′, E 34° 56.683′, with an elevation of 294 m.(Figure 1C) The Amir Formation is primarily composed of white, friable, fine‐to‐medium‐grained quartz kaolinitic sandstone, typically covered by a yellow‐brown patina (Weissbrod and Sneh 1997). Geologically, it is formed of the Lower Cretaceous Amir Formations (Figure 2D,E). The top of the Amir Formation in the northwestern area of Timna has historically been exploited for copper (Beyth et al. 1999).

## 2 Methods

### 2.1 Climatic Characterisation

#### 2.1.1 Microclimatic Characterisation of the Site Environment

From 1996 to 2000, a Campbell data logger was operated at our study site at the top of a sandstone hill (GPS N 29° 46.225′, E 34° 56.683′) (Figure 1C). Uninterrupted data were collected, encompassing five complete winter‐rain seasons at the site. Air temperature and relative humidity were measured using a Campbell 207 probe (Figure 2A). Dew was recorded with a Campbell model 237 leaf wetness sensor placed upward on the surface rock (Figure 2D). This sensor measured 102 × 58 × 58 mm with a 0.5 mm electrode gap. Photosynthetically active radiation was recorded with a Li‐Cor 190 quantum sensor (SN Q14273) configured for use with a Campbell data logger and used with the manufacturer's calibration of 4.41 microamps per 1000 μmol s^−1^ m^−2^. The surface temperature of the rock was measured using a *T*‐type thermocouple. Moisture within the upper surface of the rock was monitored at six locations by measuring the conductivity of the rock using the method described by McKay (2016). Briefly, electrodes were placed into small holes drilled 0.5 cm vertically into the rock with a separation of about 1 cm. Alternating Current (AC) excitation was used with a reference resistor of 2.2 kΩ. Conductivity values are reported in units of μ℧. Figure 2D shows the dew sensor and Figure 2C shows one of six conductivity sensors. The system recorded all sensors once every ½ h for the first 41 days and then once every ¾ h for the remaining period. All sensors were connected to a Campbell CR10 logger. The entire dataset is included in the supplemental online section (Table S1).

#### 2.1.2 Rainfall Measurements

Rainfall measurements were performed at various spatial scales and over different time frames. The spatial scales ranged from a local scale at Timna Park to a broader scale, encompassing the entire Arava region. The timeframes included daily rainfall measurements and the total annual rainfall.

##### 2.1.2.1 Daily Measurements on a Local Scale

As part of the microclimatic measurements described above (paragraph 2.1.1), the rain was measured with a Campbell TE525 tipping bucket rain gauge with a resolution (1 tip) of 0.25 mm from 7 September 1996 to 21 March 2000, encompassing three complete winter rain seasons at the site. Between 2015 and 2023, daily rain measurements in Timna Valley were carried out during eight rainy seasons using a Tipping Bucket Rain Gauge with a resolution (1 tip) of 0.2 mm (Model TB4, HyQuest) (Figure 2). The accuracy of the TB4 is ±2% for flow rates of 0–250 mm h^−1^ and ±3% for rates of 250–500 mm h^−1^ (HyQuest Solutions Pty Ltd., 2014). The Tipping Bucket Rain Gauge operated continuously throughout this period.

##### 2.1.2.2 Differences in the Regional Scale Using Average Annual Rainfall Measurements

Annual rainfall measurements were collected across the Arava Valley in southern Israel, spanning from Eilat in the south to the southern Dead Sea in the north, over eight rainy seasons between September 2015 and June 2023. Data were collected using 30 small rain gauges, four meteorological stations that are operated by the Israel Meteorological Service (IMS), and two tipping bucket rain gauges, totalling 36 measurement locations. The data was collected as part of the Rainfall and Floods Monitoring Program conducted by the Desert Floods Research Center at the Dead Sea and Arava Science Center, Israel. The data was published in annual reports and are accessible at https://floods.org.il.

##### 2.1.2.3 Inter‐Annual Rainfall Variability Based on Long‐Term Data

Inter‐annual rainfall variation was analysed using long‐term datasets obtained from the Israeli Meteorological Services data‐base (https://ims.gov.il/en/data_gov). Data was collected from two stations in southern Arava, Eilat and Yotvata. The Eilat station, located 25 km south of Timna, has annual rainfall records dating back to 1949, whereas the Yotvata station, situated 17 km north of Timna, has records starting in 1977.

### 2.2 Rock Sampling

**FIGURE 3:**
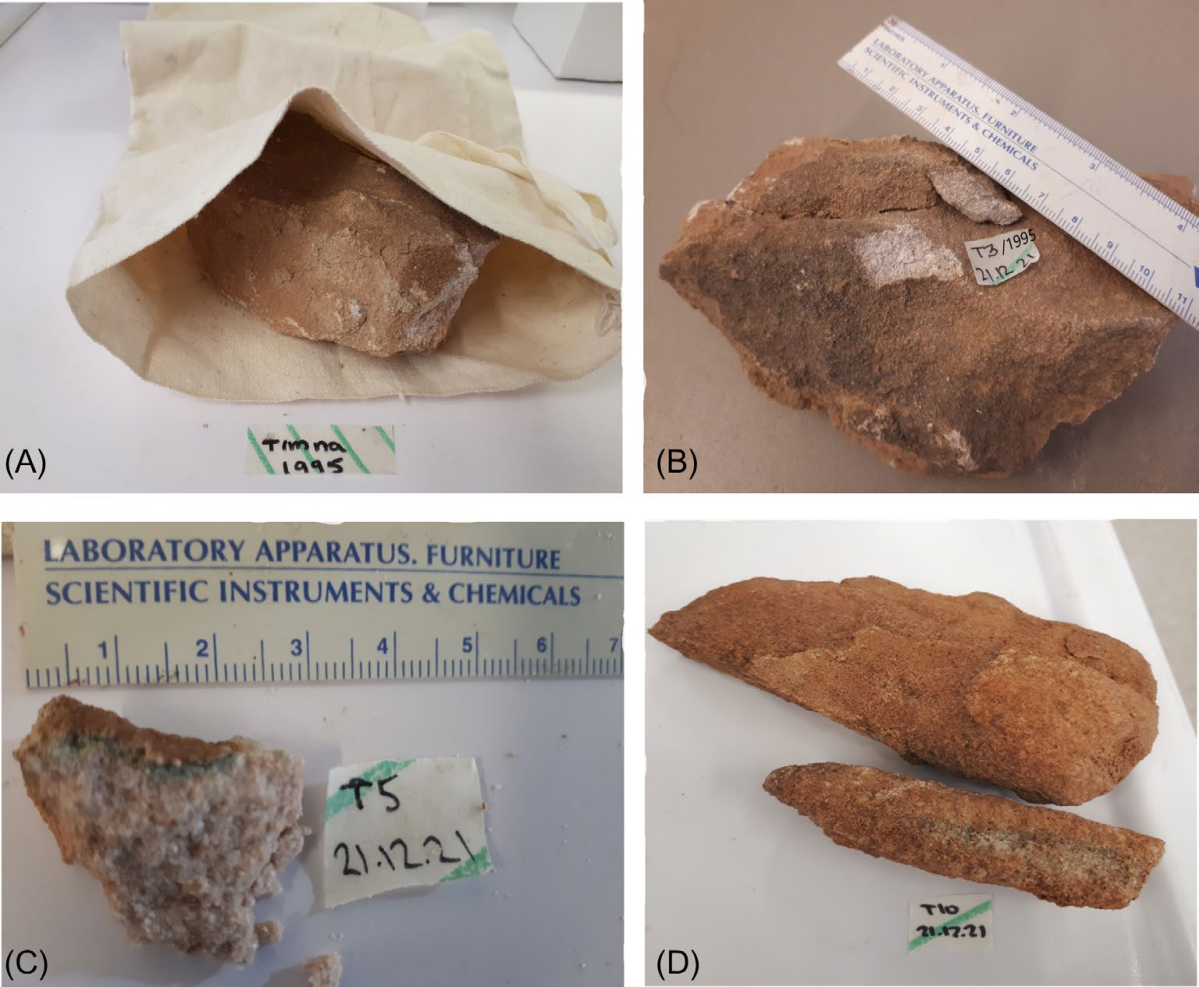
(A) Stone from 1995 that was stored in a cotton geological collecting bag till 2021. (B) Sample T3 was obtained from the stone that was collected in 1995. (C, D) Samples T5 and T10 obtained from the sandstone hill in 2021 (all sample details are shown in Table S11).

Two sets of samples from the study site were used for the examination. In 1995, samples of sandstone (~10 × 20 cm) were collected and transferred to NASA's Ames Research Center laboratories, USA, and were stored in a cotton geology bag in a dark closet at room temperature. In 2021, after 25 years, one of the stones was transferred to the environmental microbiology laboratory at Ben Gurion University for further analysis (Figure 3A,B). From this stone, four slabs of approximately 2 × 2 cm were obtained. In 2021, six additional sandstone rock samples (~10 × 15 cm) were collected from the same location. The stones were transferred with an icebox to the Environmental Microbiology Laboratory of Ben Gurion University. In the laboratory, slabs of approximately 2 × 2 cm in size were obtained using a hammer and chisel that had been surface‐sterilised with 70% ethanol (Two of them are shown in Figure 3C,D). The rock samples were divided into four subgroups for further examination. 1. Molecular investigation and DNA extraction 2. Mineralogical characterisation 3. Isolation 4. Microscopic study. For the scanning electron microscopy study, the dry samples were transferred at room temperature to the laboratory at the National Museum of Madrid, Spain, for each subgroup, containing both samples from the 1995 stone and samples from 2021. (A scheme of the study plan and the experiments is presented in Table S11).

### 2.3 Physical and Chemical Characterisation of the Rock Substrate

#### 2.3.1 Pore Analysis

The pore volume and area distribution of a representative piece of sandstone were measured by using a mercury intrusion porosimeter. (Washburn 1921; Diamond 1970) performed by Micromeritics (Norcross, GA). The surface tension of mercury is 15 times that of water. The Micromeritics report (Table S2) lists the intrusion test parameters. Briefly, mercury was forced into a representative 7.5 g sample over a pressure range that sampled pore size diameters from 344 to 0.003 μm.

The Kelvin Equation was used to determine the water activity, *a*_*w*_, in equilibrium for pores of a given size, assuming a concave meniscus of water with a spherical shape and radius equal to the pore radius *r* (e.g., Camuffo 2014, Chapter 5, 167):

(1) $$\ln\;\left(a_{w}\right)=2\;\sigma \;\mathrm{Vm}/\left(\mathrm{rRT}\right)$$

which can be related to water potential per unit volume of water, *Ψ* = (*RT/Vm*) ln (*a*_*w*_) by

(2) r=2σ/Ψ=151kPa/Ψ

where *σ* is the surface tension of water, 75.6 × 10^−3^ N/m at 0°C, *Vm* is the molar volume of water (i.e., *Vm* = 18 cm^3^ = 18 × 10^−6^ m^3^ for pure water), *R* is the gas constant (8.314 J/K mol), and *T* is the absolute temperature. The water potential was in the MKS units of Pascals (Pa).

#### 2.3.2 Rock Drying Experiments

To determine the relationship between RH and water contained in a rock as it dries, the rock sample and sensors were placed in an airtight 2.6 L chamber as described by McKay et al. (2024) until equilibrium was reached, typically a few days. The rock was then removed, and its mass was determined. This was repeated as the rock dried from saturated to air‐dried. The oven‐dried mass was determined by heating the samples to 90°C for 24 h. The saturated water mass was determined after the sample was submerged in water and vibrated slightly for 10 min. Qu et al. (2020) determined the water content by soaking the samples in water for 3 days. We found that soaking for 4 days increased the water content by 0.7% compared to our soaking for 10 min with slight vibration. This is larger than the STD of measurements (0.3%). However, we suggest that 10 min of soaking is more typical for a rain event.

#### 2.3.3 Chemical and Mineral Composition Analysis

X‐ray diffraction (XRD) analysis was performed to characterise the mineral components within the rock samples. Samples were mounted for X‐ray analysis on a front‐loading quartz XRPD sample plate and hand‐ground using an agate mortar and pestle on an as‐needed basis. The data were collected using a Panalytical Empyrean II powder diffractometer (*K* radiation, *λ* = 1.541Ǻ) equipped with an X'Celerator linear detector operated at *v* = 40 kV and *I* = 30 mA. The initial phase identification analysis was performed using the Match! Phase identification software version 2.1.1 in conjunction with the International Center for Diffraction Data (ICDD) Powder Diffraction File (PDF‐4+) database (2022 release).

#### 2.3.4 Grain Size Distribution Analysis

A sieve test was performed to determine the distribution of the sandstone grain size. A representative sample of rock obtained from the study site was ground using an agate mortar and pestle. Then, approximately 4 g of the ground stone sample was shaken through a series of sieves stacked on top of each other with decreasing mesh sizes (ranging from 355 to 125 μm) for 10 min, with the sample placed on the top sieve. Sieving was performed until the point at which the sample mass on each sieve reached a constant mass. Then, the weight of each fraction was calculated in weight percentage, giving a mass‐based distribution.

### 2.4 Characterisation of the Litho‐Biont Communities

#### 2.4.1 Scanning Electron Microscopy (SEM) Study

Fragments of colonised sandstone samples from the 1995 and 2021 collections were processed and visualised using the “SEM‐BSE technique” (Wierzchos and Ascaso 1994). Briefly, colonised rock fragments were fixed in glutaraldehyde (3% v/v) and osmium tetroxide solutions (1% w/v), dehydrated using a graded ethanol series (from 30% to 100% v/v), embedded in LR White resin, and finely polished. Blocks of embedded resin samples were finely polished, carbon‐coated, and examined with a FEI INSPECT scanning electron microscope using the backscattered electron mode (Scientific‐Technic Service of non‐destructive techniques of MNCN‐CSIC).

#### 2.4.2 Cultivation and Identification of Cyanobacteria Strains

The endolithic microbial community was scratched off the rock samples using sterile forceps on a laminar flow bench. Fragments (1 × 2 cm) from each sample (T‐1995 and T‐2021) were inoculated using sterile liquid media in Erlenmeyer flasks containing BG11 Broth (HIMEDIA manufactures) (NORMAL 9/88) and exposed to natural daylight at room temperature. Enriched cultures were examined under a light microscope, and DNA was extracted to confirm the identity of the enriched cyanobacteria. The bacterial culture was centrifuged for 1 min at a maximum speed (13,000 × g) and the resulting precipitate was used for DNA isolation using a MoBio UltraClean isolation kit (MoBio Laboratories Inc., Solana Beach, CA), following the manufacturer's protocol. The 16S rRNA sequences were amplified using a Biometra Tone thermo‐cycler PCR (Biometra, Göttingen, Germany).

The following primer sets were implemented: 106 F (5′‐CGGACGGGTGAGTAACGCGTGA‐3′) and 781 R (5′‐GACTACTGGGGTATCTAATCCCATT‐3′) (Nübel et al. 1997). Libraries were prepared from 200 ng of each sample (four samples) using the Rapid Barcoding Kit 24 V14 (Oxford Nanopore Technologies, SQK‐RBK114.24), following the manufacturer's recommendations. Sequencing was performed using MinION Mk1B (Oxford Nanopore Technologies) with 14 chemistry flow cells (R10.4.1). [High‐Accuracy/Super‐Accurate] calling was performed post‐sequencing using MinKNOW software.

##### 2.4.2.1 Data and Phylogenetic Analysis

Sequencing reads from each sample were aligned against the known 16S reference sequences of *Chroococcidiopsis* using BLASTn. Multiple reads with base pairs (bp) that closely matched the ribosomal 16S gene were selected for further analysis.

The selected sequences from our dataset, along with the reference *Chroococcidiopsis* sequences and *Gloeobacter* as an outgroup, were aligned using the MAFFT web server with default settings. A maximum likelihood (ML) tree was constructed using MEGA version 11.0.13, and the resulting Newick tree was exported to iTOL for visualisation.

#### 2.4.3 DNA Extraction and Metagenomic Sequencing

Colonised stone samples (T2, T3, and T4 related to the 1995 samples, T5, T6, T9, and T10 related to the 2021 samples) were crushed in a laboratory hood to obtain small aggregates using a sterilised plier. Aggregates (0.5 g) from each sample were transferred into separate bead tubes for DNA extraction. DNA extraction was performed using a MoBio PowerSoil DNA Isolation Kit (MoBio Laboratories Inc., Carlsbad, CA, USA) following the instructions. The purity and concentration of DNA samples were determined using a Nanodrop ND‐1000 spectrophotometer (Nanodrop Technologies, Wilmington, DE, USA). The extracted DNA was submitted to the Genomics and Microbiome Core Facility at Rosh University, USA, for whole‐genome shotgun sequencing (WGS). DNA was processed using a Nextera XT library preparation kit and sequenced on an Illumina NextSeq500 instrument.

#### 2.4.4 Bioinformatics Analysis

Of the six samples sent for sequencing, only three (T2, T6, and T10) had sufficient reads for metagenomic analysis. For each sample, paired‐end FASTQ files (forward and reverse) were obtained. Data was processed using the NeatSeq Flow workflow platform (Sklarz et al. 2018). Initially, raw reads were examined using FastQC (Andrews 2010), and low‐quality or unwanted reads were trimmed or removed using Trimmomatic (Bolger et al. 2014) and Bowtie2 (Langmead and Salzberg 2012). Taxonomic profiles were generated using the Kaiju software package (Menzel et al. 2016) and visualised using the ggplot2 package in R (R Core Team 2013). To construct and characterise metagenome‐assembled genomes (MAGs), metagenomic data were processed using the metaWRAP pipeline. Quality control of the raw reads was performed using TrimGalore (Krueger 2015), and low‐quality reads were removed. Quality‐controlled sequences were assembled using MetaSPAdes (Nurk et al. 2017). The resulting assemblies, along with quality‐controlled sequences, were used for metagenomic binning using three different algorithms: MaxBin (Wu et al. 2014), metaBAT (Kang et al. 2015), and CONCOCT (Alneberg et al. 2013). These bin sets were consolidated to obtain a more robust set, with a minimum completion of 50% and a maximum contamination of 10%. The consolidated bin set was reassembled using both “strict” and “permissive” algorithms, and if the reassembled bin showed improvement, it replaced the original bin. Functional annotation of the consolidated bins was performed using Prokka (Seemann 2014) within the Annotate bins module of the metaWRAP. The relative abundance of the draft genomes across the samples was determined using the Quant_bin module of metaWRAP. Taxonomic identification of the metagenome‐assembled genomes (MAGs) was performed using GTDB‐Tk v2.4.0 (Genome Taxonomy Database Toolkit) (Chaumeil et al. 2022), relying on reference data version r220 of the GTDB database (Parks et al. 2020).

## 3 Results

### 3.1 Microclimatic Characterisation

**FIGURE 4:**
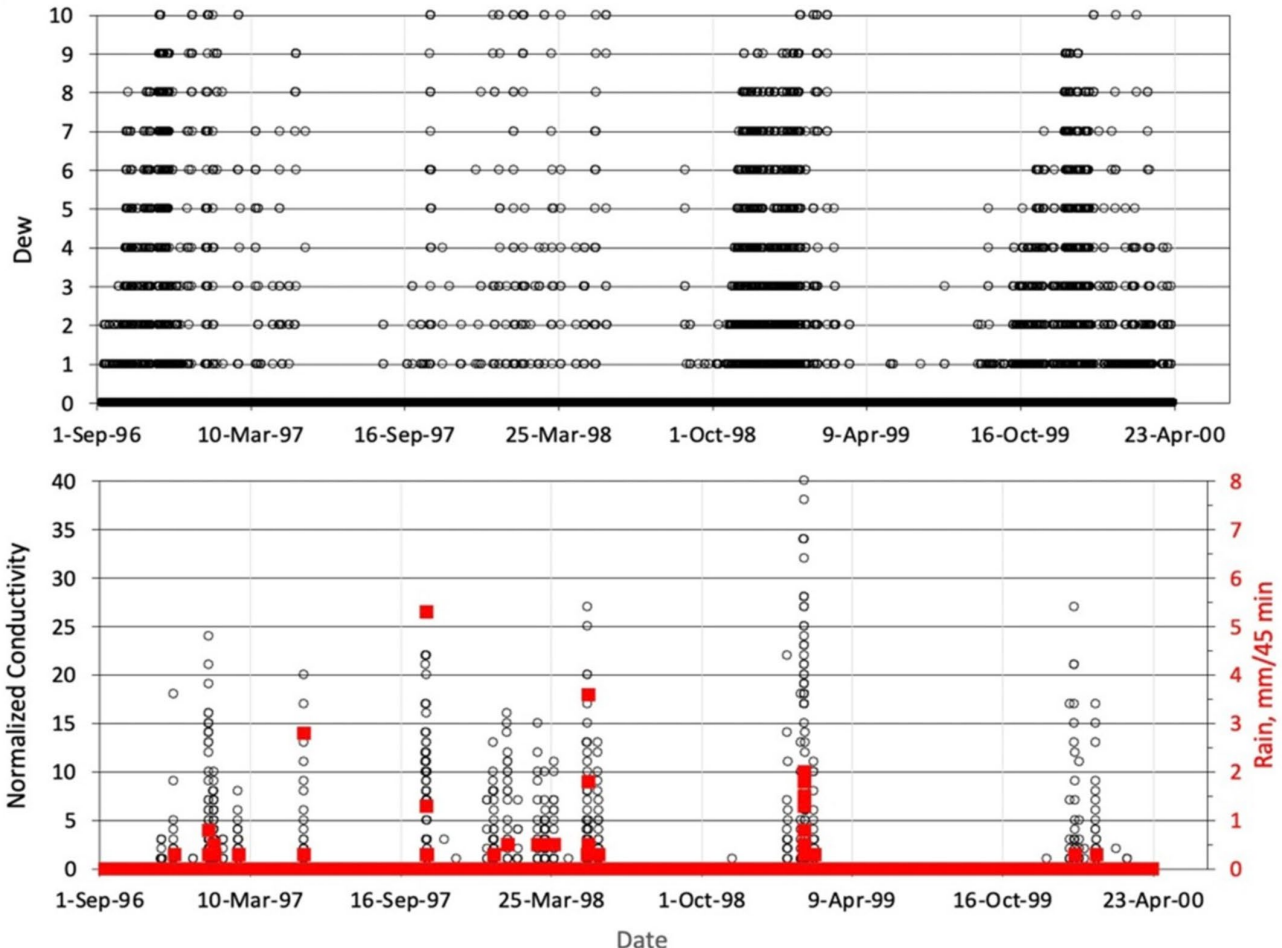
Top panel: Dew averaged over the 45‐min sampling interval. Bottom panel: Conductivity sensor #2 compared to the rain (in red) accumulated in the 45‐min sampling interval.

Figure 4 presents a summary of the meteorological data over three continuous years of operation of the microclimate station. The data are analysed over the calendar year except for rain, which is summed based on a hydrological year that begins with September of the previous year. Hence, the hydrological year of 1998 ran from September 1997 to the end of August 1998. This allows a direct comparison to other rain compilations in the Negev (Sherman 2010). As expected, the data in Table S1 indicate that the site is typically hot and dry, with a mean air temperature of ~25°C and a maximum air temperature of ~46°C. The mean relative humidity is ~26%. The maximum temperature of the rock surface over the data recording period was 56.7°C, 11.4°C hotter than the corresponding air temperature of 45.3°C. The maximum value of incident light was 2138 μmol s^−1^ m^−2^ on 14 May 1998, at 12:15. Typical summer (May, June, July–noon time values were 1950–2000 μmol s^−1^ m^−2^). The mean sunlight shows a slight but steady reduction in time of approximately 2% per year, probably due to dust buildup on the sensor. The value of 480 μmol s^−1^ m^−2^ corresponded to a total solar flux of approximately 230 W m^−2^. Notably, the microclimate record indicated that the nighttime temperature of the rock surface consistently exceeded the atmospheric dew point derived from ambient temperature and relative humidity. At its lowest temperature in the data set, the rock surface exceeds 2.3°C above the atmospheric dew point. As shown in Figure 2D, the dew sensor was a condensing plate attached to the rock surface. These sensors are likely associated with atmospheric conditions and indicate dew formation before condensation on bare soil or rock surfaces (Agam and Berliner 2004, 2006). Several instances in our microclimate data revealed that the dew sensor detected elevated dew levels, whereas the rock conductivity measurements indicated the presence of liquid water on the rock surface but no recorded rainfall. However, the fact that the surface temperature exceeds the dew point may indicate that what we are recording is a slight rain that is below the measurement threshold of the tipping bucket rain gauge (0.25 mm total in the sampling interval of 45 min) and not condensation of liquid water on the rock surface. In this case, the condensing plate of the dew sensor acts as a highly sensitive rain indicator (Figure 2B).

### 3.2 Rainfall Measurements Along the Arava and Timna Valley

An overview of the entire dataset for rain, dew, and conductivity (sensor #2) is presented in the (Table S1). The results from the six conductivity sensors showed consistency in the initial response to moisture, but there was a variation in the decay of the conductivity signal as the rock dried. This variation occurred at conductivity levels below the level of interest, in terms of water availability for cyanobacteria. Sensor #2 exhibited a typical response and was used in this analysis as an example sensor.

#### 3.2.1 Daily Rainfall Measurements on a Local Scale

**FIGURE 5:**
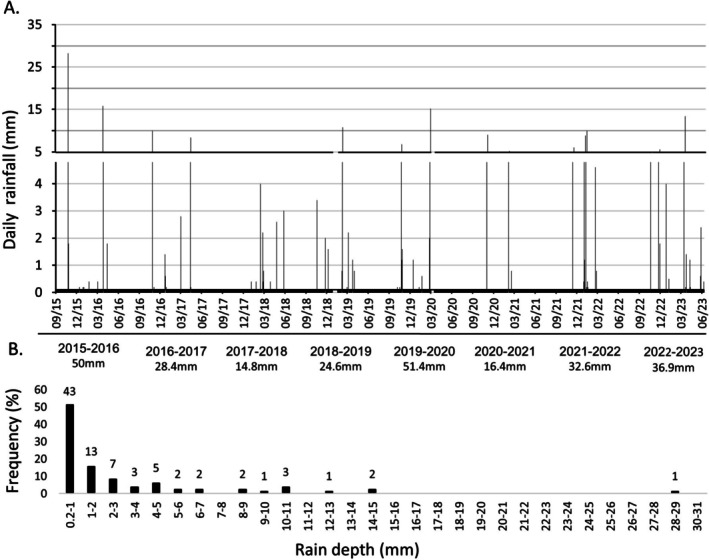
Daily and Annual rainfall in Timna Valley. (A) Daily and annual rain measurements recorded over 8 years (September 2015–August 2023). The annual rainfall is indicated below the *x*‐axis, according to the rainy season. (B) frequency distribution of daily rainfall depth. (Calculated from a total of 84 rainy days over 8 years). The numbers above the bars represent the number of days in each category. The measurement threshold and resolution (accuracy) are 0.2 mm.

Daily rainfall measurements collected over eight rainy seasons indicated that the mean annual precipitation was 31.9 mm, ranging from 14 to 51 mm (Figure 5A). During this period, 84 rainy days occurred, with an average of eight rainy days per year. The frequency distribution of daily rainfall depth revealed that 51% of days recorded less than 1 mm of rainfall, 23% recorded 1–3 mm, 12% recorded 3–6 mm, and only 7 days (14%) recorded more than 6 mm, with daily totals ranging from 6 to 28 mm (Figure 5B). There was considerable variability in the onset and cessation of the rainy season between years. Although rainfall generally occurs between October (autumn) and May (spring), it has been observed to start as late as 5th January (in 2018) and end as early as 17th February (in 2021). The dry period lasted an average of 216 days, with a maximum of 266 days between 14 April 2017, and 5th January 2018.

#### 3.2.2 Regional Rainfall Measurements on a Regional Scale

**FIGURE 6:**
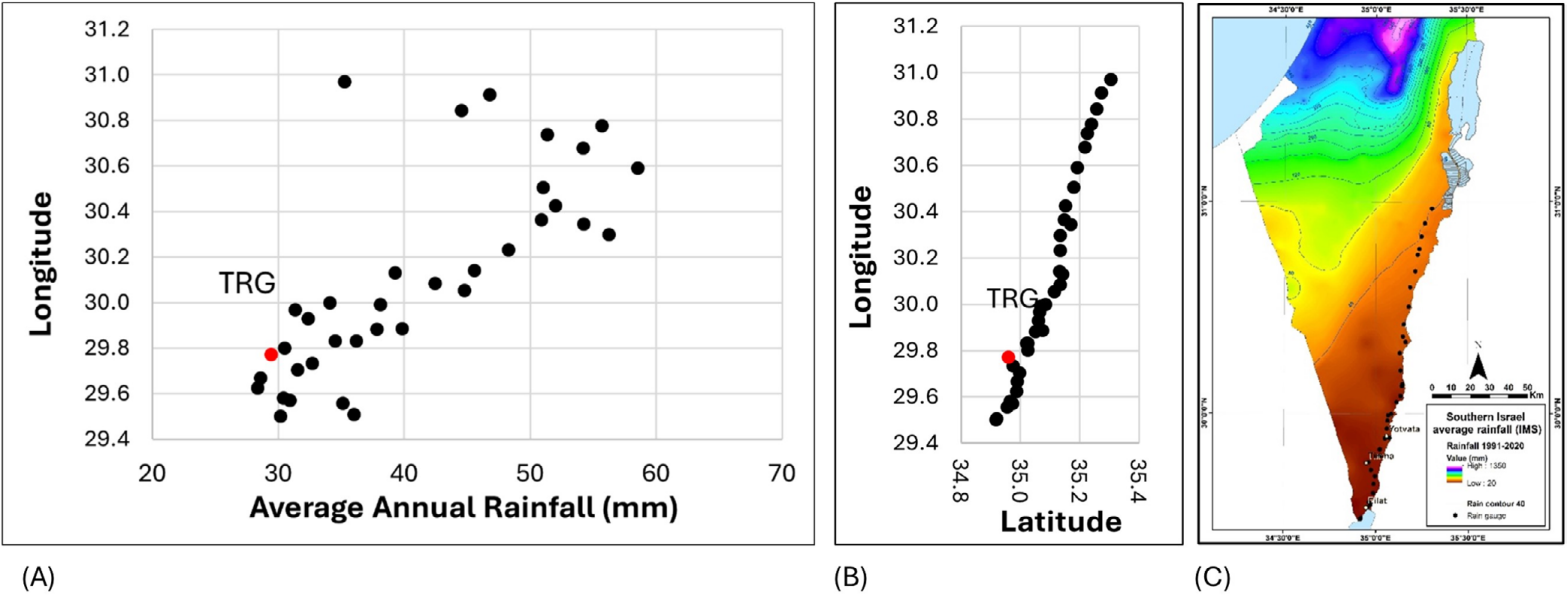
Changes in annual rainfall at a large spatial scale. (A) Mean annual rainfall during the hydrological year (Sept—Aug) across the Arava Valley represents 6 rainy seasons between 2017 and 2023. (B) Rain gauge's location across the Arava. Data points represent 36 rain gauges from south to north, showing the Latitudes and Longitudes scale (Geographic coordinates, WGS84 datum). The Timna Rain Gauge (TRG) is marked in red. (C) Map of southern Israel's average rainfall based on average annual rainfall between 1991 and 2020 (The data was obtained from the Israel Meteorological Service (IMS 1949)), (https://ims.gov.il/en/data_gov). Black dots represent the positions of rain gauges.

Arava Valley and southern Negev are the driest areas in Israel. Generally, the mean annual rainfall between 2017 and 2023 increased from south to north along Arava (Figure 6). The Timna rain gauge (TRG) recorded the third‐lowest rainfall level in the region. Rain gauges located near the northern tip of the Gulf of Aqaba (Eilat area) in the southernmost part of the region show slightly higher precipitation than those in the northern areas of southern Arava. Additionally, sampling points near the Dead Sea exhibited lower rainfall levels than those in central Arava.

**FIGURE 7:**
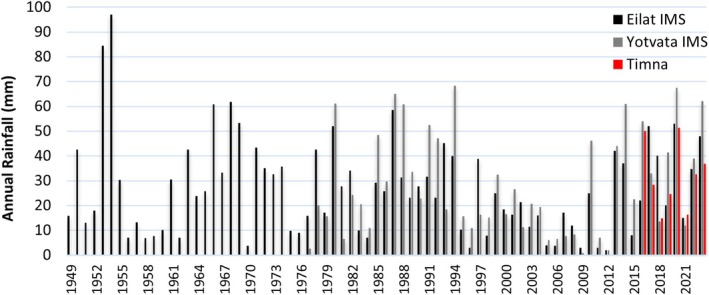
Inter‐annual rainfall in three stations across the Arava. Long‐term data sets from Eilat (25 km south of Timna) and Yotvata (17 km north of Timna) were obtained from the Israeli Meteorological Services (IMS), while the Timna data set was recorded during the current study.

Inter‐annual variation in rainfall over long timescales highlights significant variability across decades of measurements, with annual rainfall ranging from 1 to 97 mm. Data from Eilat (since 1949, 25 km south of Timna) and Yotvata (since 1977, 17 km north of Timna) revealed consecutive years with near‐zero rainfall. For example, between 2005 and 2009 and between 2011 and 2012 (Figure 7).

### 3.3 Physical and Mineralogical Characterisation of the Rock Substrate

#### 3.3.1 Pore Size Distribution Analysis

**FIGURE 8:**
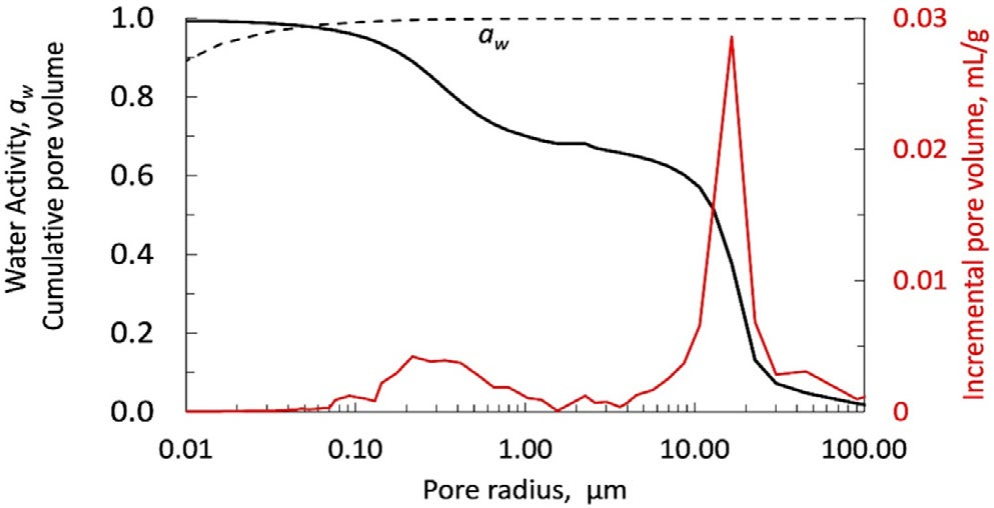
Incremental pore volume as a function of pore radius (red line and red axis). Water activity (*a*_*w*_) as a function of pore radius (dotted line). Normalised cumulative pore volume (solid line). Over 98% of the pore volume corresponds to *a*_*w*_ > 0.98.

Figure 8 shows the pore size distribution determined from Hg infusion (Table S2) and the integration of this distribution from the largest pore down to the smallest. The larger the pore size, the higher the *a*_*W*_ value associated with that pore. For a pore radius of 0.053 μm, the value of *a*_*w*_ is 0.98 (RH = 98%), and 98% of the pores have a radius larger than 0.053 μm.

#### 3.3.2 Rock Drying Experiments

**FIGURE 9:**
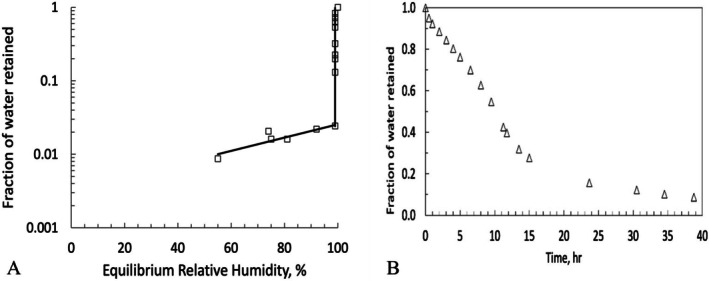
(A) Fraction of water retained in Timna sandstone rock 1 (mass 0.465 kg) as a function of the equilibrium relative humidity of the rock when equilibrated with sensors in a closed chamber. (B) Drying curve for Timna sandstone of dry mass 0.465 kg from the same larger rock used for pore analysis. The sample was soaked in water for 15 min and then dried at 22°C, 55% RH. The plot shows the water mass in the rock normalised to the saturated water amount in the same rock.

Table 1 and Figure 9 display the outcomes of the rock drying studies, demonstrating the water retention in the Timna sandstone.

**TABLE 1:** Summary of rock properties for Timna sandstone rock.

Property	Value	Note
Hg intrusion volume	0.0638 mL/g	Supporting Information
Saturated wet mass, rock	485.59 g	Measured
Oven dry mass	463.65 g	Measured
Saturated water volume	0.047 mL/g	Calculated

#### 3.3.3 Chemical and Mineral Composition

XRD patterns indicated that Quartz (Silicate Oxide) is the predominant mineral in the Timna sandstone rocks. Kaolinite (Aluminium Silicate Hydroxide) was identified in all samples, albeit in relatively minor quantities. Furthermore, Calcite (Calcium Carbonate), Paragonite (Sodium Aluminium Hydroxide), and Iron Hydroxide were present in some samples, also in low concentrations. XRF analysis revealed that Silica (Si) is the principal element in all sandstone samples, comprising 76%–87.7%, followed by Aluminium (Al) at 6.9%–11.9%, Calcium (Ca) at 0.87%–10.2%, Iron (Fe) at 0.3%–1.5%, and Titanium (Ti) at 0.3%–1%. Other elements were detected in quantities less than 0.5% (Table S5).

#### 3.3.4 Grain Size Distribution

Table S4 presents the results of the grain size analysis. The particle size distribution was mostly settled within the 250‐ and 180‐μm sieve sizes, which corresponded to the fine–coarse sand fractions.

### 3.4 Characterisation of Lithobiontic Communities

#### 3.4.1 Scanning Electron Microscopy Study of Colonised Sandstones

**FIGURE 10:**
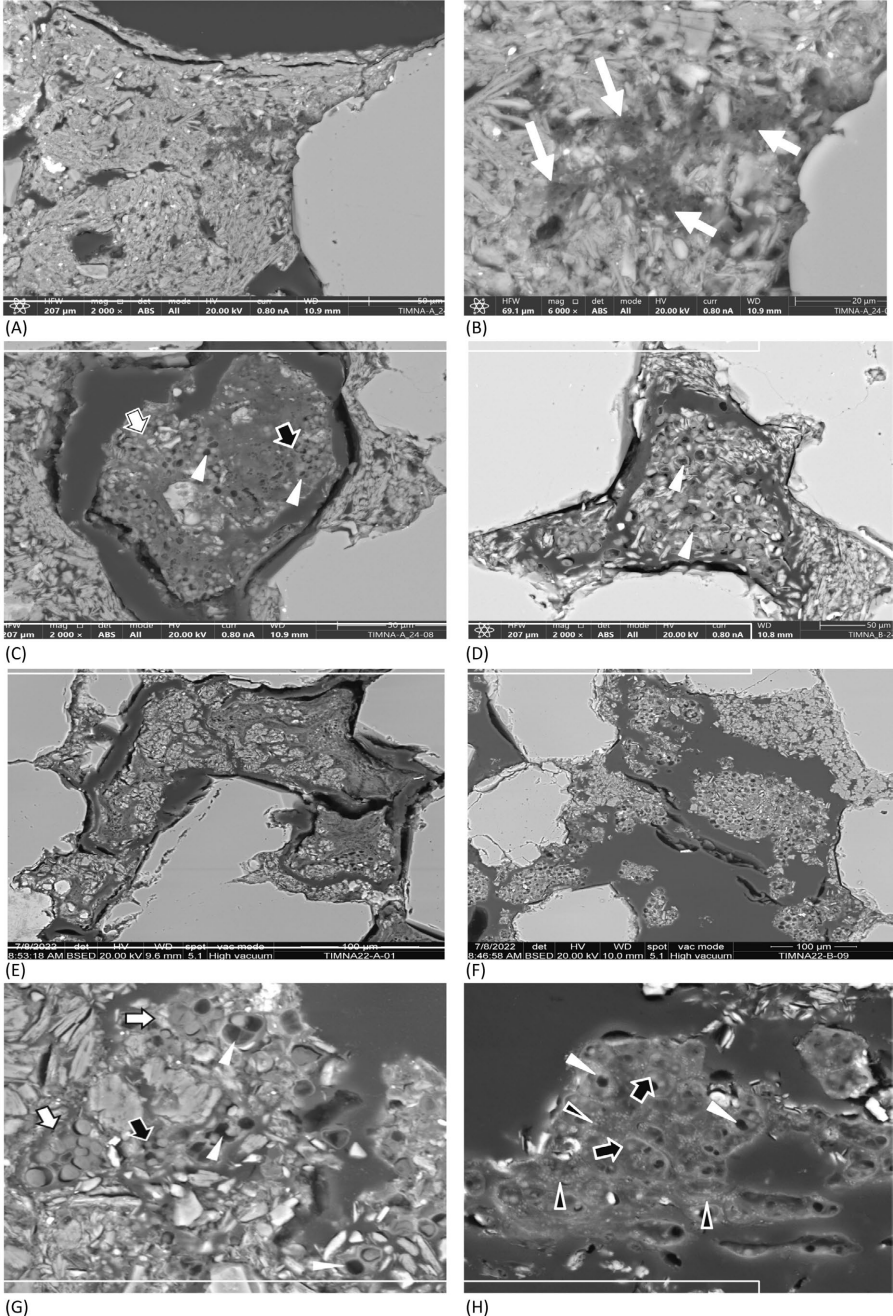
SEM‐BSE images of endolithic communities from Timna sandstones collected in 1995 (A, C, E, G) and 2021 (B, D, F, H). (A): Heterotrophic bacteria embedded in a mineral matrix formed near the surface. (B): Magnified view of the area marked by the square in image (A), showing a heterotrophic bacterial colony (indicated by arrows). (C, D): Communities dominated by cyanobacteria intermixed with mineral fragments within sandstone pores of the samples collected in 1995 (C) and 2021 (D), containing both dead and living cyanobacteria cells. (E, F) Clusters of cyanobacteria with numerous dead cells in samples collected in 1995 (E) and 2021 (F), respectively. (G): Magnified view of cyanobacterial aggregate types, including groups of densely packed, irregularly arranged cells surrounded by a common envelope (black arrow), and groups of 2–4 larger cells enclosed by a thin shared envelope (white arrows). White arrowheads indicate dead cells. (H), endolithic community showing heterotrophic bacteria associated with cyanobacteria remnants (black arrowheads).

A similar colonisation pattern was observed in samples from 1995 to 2021. Close to the surface, heterotrophic bacteria were embedded in a mineral matrix rich in silicon and aluminium (Figure 10A). Small bacterial cells (white arrows in Figure 10B) are observed in association with remnants of bacterial cells that lack cellular content but retain their envelopes, forming a dense organic network (Figure 10A–D). Beneath this initial layer, dominated by heterotrophic bacteria, the pores between the quartz grains of the sandstone were filled with clusters of cyanobacterial cells (arrows in Figure 10C), exhibiting increased BSE signal due to the presence of thylakoids in the cytolasm, and intermixed with mineral fragments (asterisks in Figure 10C,D). In certain areas, these clusters contained numerous dead cells (Figure 10E,F), showing only remnants of the cyanobacterial cell wall (white arrowheads in Figure 10C,D).

Two distinguishable morphotypes of cyanobacteria were prevalent in pores harbouring microbial communities dominated by cyanobacteria in the sandstones collected during both periods (Figure 10C–H). Some pores were primarily occupied by small groups of cells in microscopic solitary colonies, usually consisting of 2–4 cells surrounded by a common thin envelope (white arrows in Figure 10C,G). These cells were predominantly spherical or hemispheric and were approximately 4–6 μm in diameter. In other pores, these clusters also contained microscopic colonies with densely irregularly arranged cells surrounded by a common envelope (black arrows in Figure 10C,G). The cells were spherical and approximately 2–2.5 μm in diameter, displaying features typical of *Chroococcidiopsis* cells. Dead cells were common in both cyanobacterial morphotypes (white arrowheads in Figure 10C,D,G). Heterotrophic bacteria were commonly found in areas where dead cyanobacterial cells were abundant (black arrowheads in Figure 10H).

#### 3.4.2 Cultivation and Identification

**FIGURE 11:**
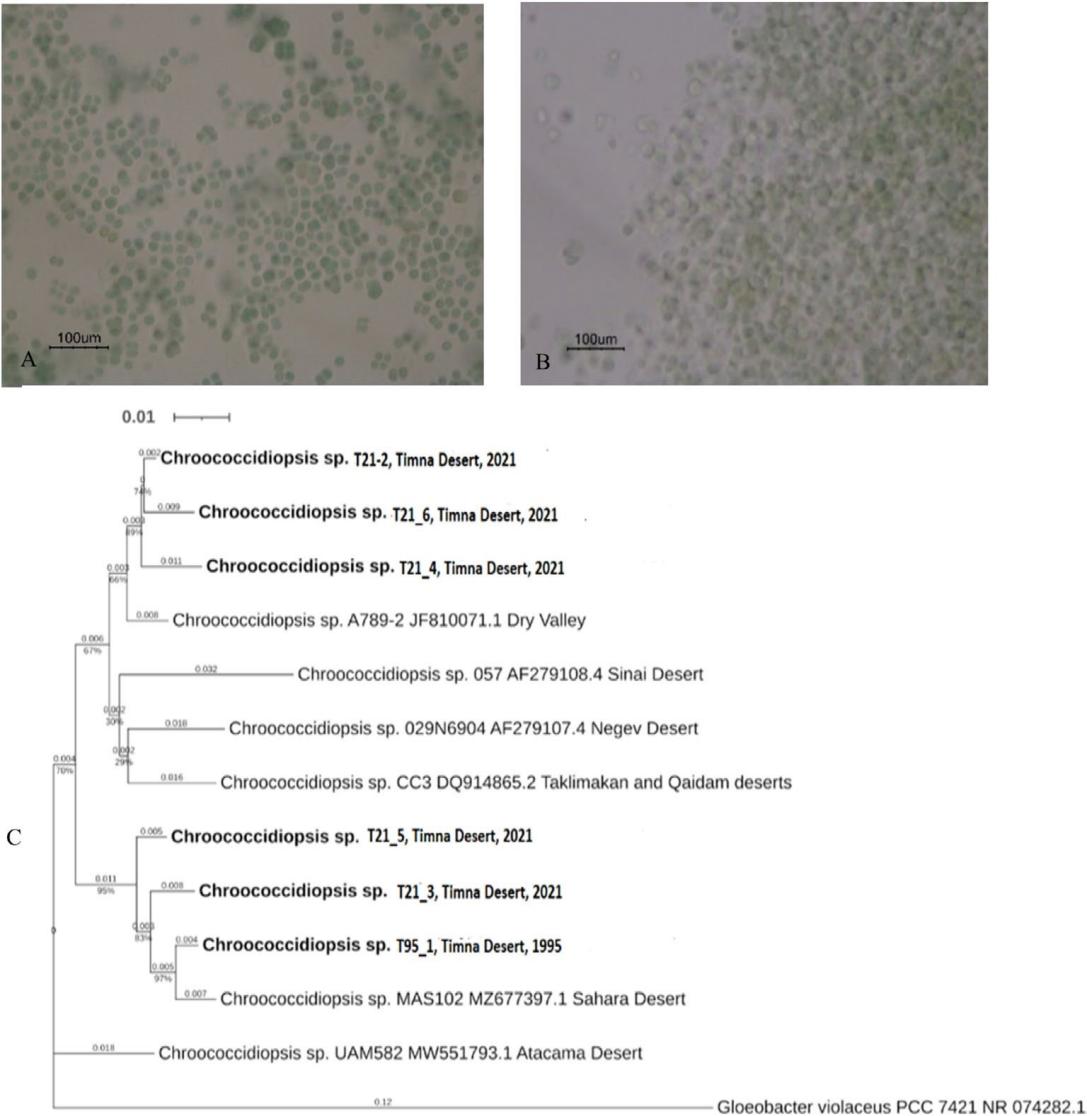
Light microscope images of Cyanobacteria enrichment cultures from Timna's sandstone samples obtained in 1995 (A) and 2021 (B). (C) Phylogenetic relations of bacterial 16S rRNA gene sequences obtained from 1995 and 2021 enriched cultures (in bold) and closely related sequences from the NCBI database. Horizontal lengths are proportional to the evolutionary distance. Bar = 0.01. The numbers above each node represent bootstrapping statistical results. *Gleobacter violaceus* is used as an outer group.

The cultivation of cyanobacteria from the sandstone samples resulted in non‐axenic cultures dominated by dark‐green unicellular bacteria surrounded by a colourless sheath. The cells typically formed spherical or irregular clusters, with an average diameter of 2–6 μm (Figure 11A,B), DNA extraction and Nano‐pore sequencing technology were used to identify the dominant taxa. A BLASTX N database search revealed that most of the sequences (> 90%) were clustered in the *Chroococcidiopsis* genus. There were two clusters (Figure 11C, Table S10): a group of both sequences obtained from Timna 1995 samples and sequences obtained from 2021, which shared > 93% similarity with *Chroococcidiopsis* sp. MAS102 (Mehda et al. 2022). The other cluster included sequences obtained from Timna 2021 samples and showed > 93% similarity to *Chroococcidiopsis* sp. A789‐2 JF810071.1, obtained from Dry Valleys, Antarctica.

#### 3.4.3 Metagenomic Analysis

**FIGURE 12:**
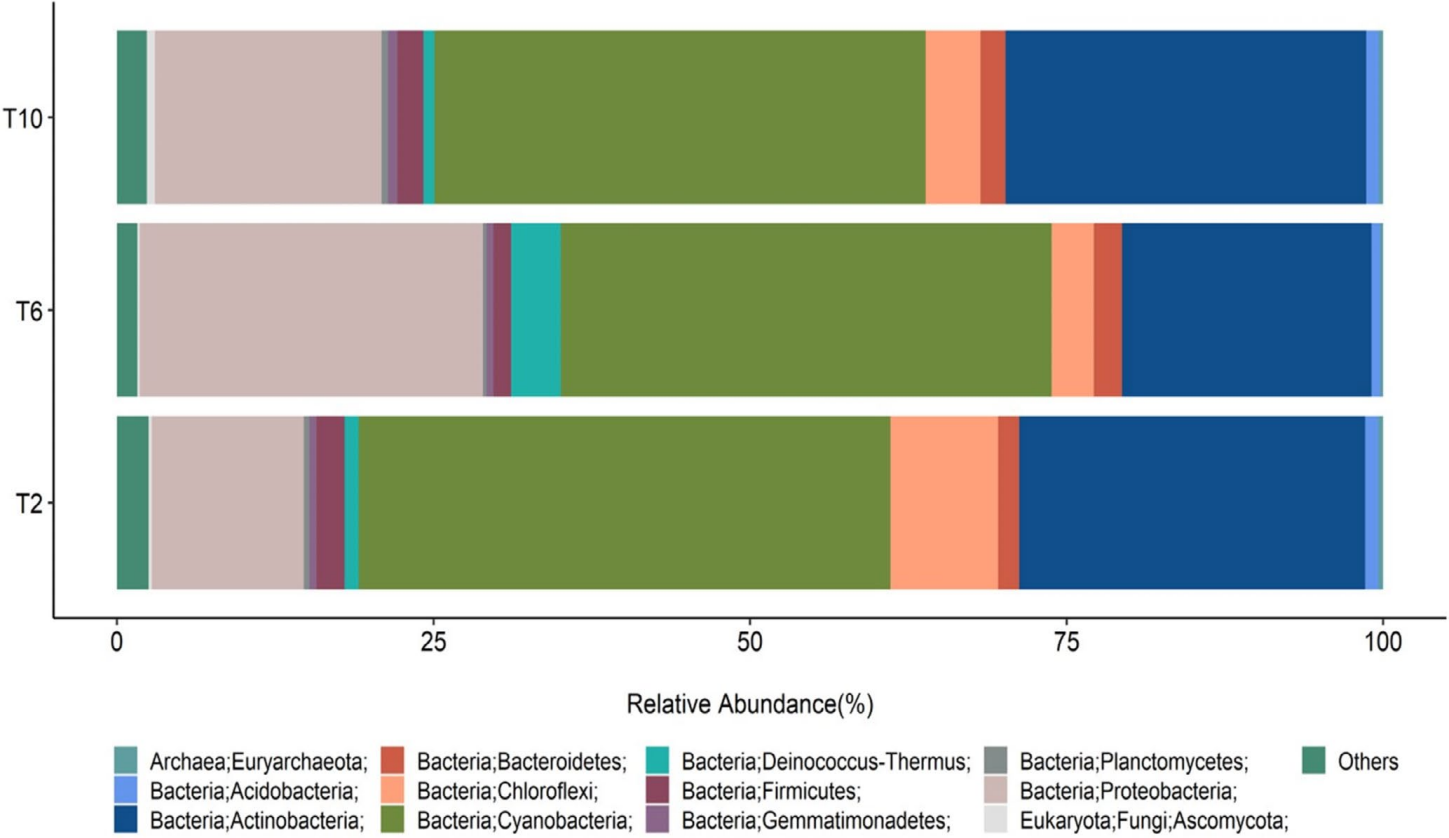
Relative abundance of bacterial phylum in endolithic communities from Timna sandstone samples obtained in 1995 (T2) 2021 (T6, T10) using shotgun sequencing analysis.

The results presented in Figure 12 provide insights into the community structure of the microbial communities associated with sandstone rock samples using shotgun analysis. In general, about 95% of the sequences obtained from all the samples were phylogenetically assigned to the bacterial domain. Only a small fraction was assigned to the Archaeal (0.5%–1%) and the eukaryotic domains (1%). Among the Bacterial taxa, Cyanobacteria was found to be the dominant phylum (30%–40%), followed by Actinobacteria (20%–30%). Other phyla found were Chloroflexi (4%–10%), Deinococcos thermos (2%–5%), Gemmatimonadetes (1%–2%), Firmicutes (2%–3%), Bacteriodetes (2%–3%), and Acidobacteria (1%–2%). Within the Cyanobacteria phylum, members of the class Nostocales were the most dominant (1.08%–12.8%), represented mainly by the genera *Nostoc*, *Scytonema, Calothrix*, and *Tolypothrix. The class* Chroococcidiopsidales (4.8%–5.4%) was represented by the genera *Aliterella* and *Chroococcidiopsis* (Tables S3 and S6).

#### 3.4.4 Metagenome‐Assembled Genomes (MAGs)

DNA sequences from the shotgun metagenome analysis were assembled into 24 bins, all with completeness greater than 50% and contamination less than 10% (Table S7). GTBK taxonomic annotation allowed the identification of 24 MAGs representing potential bacterial strains found in the studied environment. Among these, seven bins represent the cyanobacterial family Chroococcidiopsidaceae. Bin 17 (96% completeness) was found to be the most abundant of all samples. Bin 2 and Bin 12 were annotated to the genus *Chroococcidiopsis*. Bin 2 was found with 99.03% completeness and 0.44% contamination. The metagenome‐assembled genome size was found to be 5,569,123 bp, with an N50 72038 and GC content of 0.43 (Table S8). Therefore, Bin 2 was further studied for the genes involved in the mechanisms of resistance to environmental stress. In total, approximately 4990 genes were identified. Table 2 presents genes related to potential mechanisms involved in desiccation tolerance (e.g., EPS, osmotic regulation), heat (e.g., chaperones, DNA repair), ultraviolet radiation (UVR) stresses, and oxidative stress (Table 2 and Table S9).

**TABLE 2:** Stress response genes revealed in BIN2 related to the *Chrooccodiopsis genus*.

Environmental stress	Genes represesnted in the genome	COG annotation
UV radiation	uvrA, uvrB, uvrC uvsE, uv	COG4294, COG0178, COG0322
Carotenoids		COG3670
Heat (chaperones)	clpB, dnak, dnaJ, htpG, surA	COG0542, COG0326
EPS	lptC, lptB, lspA, rfaQ, msmX	COG0859, COG1137
Cell wall	ftsL, mrdA, mrdB, pal, patA, cotA, hspA	COG0768, COG0772
Osmotic regulation		
Trehalose	sugA, sugB	COG1175, COG0395
Sucrose	susA	COG0438
Potasium transport	klmA, kdpA, kdpB, kdpC, nhaS, nhaP, ktrA, ktrB	
Oxidative stress		
Glutahnione	kefB, kefC	
Superoxide dismutase	sodA, sodC	COG2032
Peroxide	perR	COG0735
Catalase	katA	COG0753
Thioredoxin	trxA, trxB	COG0526
Response regulator	mprA, pleD, vraA	
SOS response‐associated protein	yedK	COG2135
DNA repair	mutL, mutS, recO, recF, recN, rcp1, radA, dps	COG0323

## 4 Discussion

In this study, we focused on the microbial colonisation of sandstone rocks in the hyper‐arid desert of the Timna Valley, Israel. We argue that the study site has a set of extreme environmental conditions that foster the survival of a distinct microbial community. Among them, we emphasise the adaptation and survival abilities of cyanobacterial taxa within this challenging habitat.

## 5 Geophysical Conditions and Water Activity of Timna's Sandstone

It is already known that the bioreceptivity or susceptibility of rocks to colonisation mainly depends on the physical and chemical properties of the rock substrate (Guillitte 1995). For example, the grain size distribution and linked pore network features of lithic substrates are essential factors that influence water activity (aw) (McKay et al. 2024). Our study demonstrates that, in Timna sandstone samples, water‐saturated rock (e.g., water activity > 0.98) takes approximately 2 days to dry. During the drying period, the water activity drops precipitously from 1 to below the usable level with virtually no intermediate water activity. This is in contrast to the similar curve for Antarctic Beacon sandstone (figure 5 in McKay et al. 2024). Although the Timna sandstone appears superficially like the Antarctic Beacon sandstone, the difference in the drying curves is striking. The RH in the Antarctic rocks begins to fall below 98% when ~10% of the pore water is still present in the rock, providing conditions that allow for lichen growth, but not cyanobacterial (McKay et al. 2024). In contrast, the RH in the Timna rocks only begins to fall below 98% when ~2% of the water is still present in the rock. There is virtually no time when the relative humidity in the Timna sandstone is too low for cyanobacteria but high enough for lichens. Thus, the ability to uptake water at lower water activity (aw), such as lichens (Kidron et al. 2023) may not be useful in Timna sandstone. Thus, the nature of pore space distribution and water retention in the Timna sandstone may be a selection factor for endolithic cyanobacteria over endolithic lichens. This also contrasts with the varying moisture conditions of halite formation in the hyper‐arid Atacama Desert. When the relative humidity of the air exceeds 70%–75%, halite will absorb moisture, a process referred to as deliquescence (Wierzchos et al. 2006).

## 6 Water Resources in Timna: Pulse Precipitation Events

Our findings on the water sources in Timna indicated consistently low air humidity and limited dew on rock surfaces during the microclimate data collection period. Consequently, the sole source of sufficient water to generate liquid on the rock surface is infrequent and typically minor rainfall episodes. Therefore, we suggest that the main water source is rainfall. Moreover, the daily and annual rainfall measurements in Timna Park revealed that rainfall is both minimal and highly variable. Eight years of rainfall data underscore the extreme variability and scarcity of precipitation in Timna, observed both daily and annually. The region experiences very few rainy days each year, with most contributing negligible amounts of rain. Substantial rainfall events are rare, and the annual totals can vary widely between years. Furthermore, the timing of the start and end of the rainy season is highly variable, whereas extended dry periods are a common feature of the climate. From this, we conclude that Timna has distinct microclimate conditions characterised by small and short‐duration precipitation events. This variability in rainfall underscores the considerable challenges faced by the living organisms in these regions. This is consistent with other arid ecosystems, in which individual events offer brief pulses of resource availability for desert organisms (Sala and Lauenroth 1982; Coe et al. 2012; Azua‐Bustos et al. 2013; McHugh et al. 2015). In these environments, water pulses are spaced between the long‐term desiccation periods. The pulse‐reserve paradigm, established by Noy‐Meir (1973), aims to elucidate the adaptation of organisms to these conditions. The emphasis was mostly on vegetation and has been incorporated into a thorough approach for examining plant diversity in dry regions (Maestre et al. 2021; Liu et al. 2023; Gross et al. 2024). Only recently, Garcia‐Pichel and Sala (2022) applied the Extended Pulse Reverse Paradigm (EPRP) to microorganisms. This approach focuses on biological soil crusts (Kut and Garcia‐Pichel 2024). The endolithic system has also been recognised as a potential model for these studies (Kut and Garcia‐Pichel 2024).

## 7 Tolerance to Long‐Term Desiccation Periods in Endolithic Microorganisms

In this study, sandstone samples that were collected several decades ago (e.g., 1995) from a sandstone hill in Timna Valley, Israel, and stored in a cotton geological bag in a dark closet for approximately 20 years, were studied alongside samples obtained from the same site in 2021. Interestingly, all the samples exhibited a distinct blue‐green layer of photosynthetic microorganisms located 0.5–5 mm below the rock surface. Molecular analysis, along with laboratory cultivation procedures, revealed a mixed bacterial community dominated by cyanobacteria. These findings align with those of previous studies conducted at the same site (Friedmann et al. 1967; Friedmann 1980; Weber et al. 1996). It is well known that cyanobacteria are difficult to purify and grow in axenic cultures (Rippka et al. 1979; Cornet et al. 2018; Ramos‐Barbero et al. 2019). Consequently, obtaining cyanobacterial DNA without contaminating the sequences is a challenging and time‐consuming process. Nevertheless, Nanopore sequencing technology targeting the 16S rRNA gene revealed most sequences related to the genus *Chroococcidiopsis*. Interestingly, aligning the sequences obtained from the 1995 and 2021 samples indicated a high percentage of similarity. As shown above, these cyanobacterial‐dominated communities could withstand long periods of desiccation. For example, it was found previously that the cyanobacterial genus *Chroococcidiopsis* can enter a state of suspended animation, resuming metabolism upon rehydration (Billi and Potts 2002; Billi 2009; Wierzchos et al. 2012; Li et al. 2022; Baldanta et al. 2023). Our SEM‐BSE analysis revealed that healthy and dead cyanobacterial cells were intermingled within the communities inhabiting the sandstone pores in samples collected in 1995 and 2021. This observation indicates that only a subset of cyanobacterial cells is capable of persisting. Such physiological heterogeneity has been previously documented in cyanobacterial‐dominated communities from other extreme environments, such as Antarctica (De los Ríos et al. 2004; Wierzchos et al. 2004) or the Atacama Desert (Wierzchos et al. 2015), and is considered an ecological advantage. Leakage and lysis products from dead cells may support the growth of surviving cells. These communities contain numerous remnants of dead cells that form an organic matrix in which living cells are embedded, consistent with observations from lithobiontic microbial communities in other hyper‐arid desert regions (Wierzchos et al. 2015; Nir et al. 2023). Furthermore, the close interactions observed between cells at different physiological stages as well as between various microbial components, such as the association of heterotrophic bacteria with cyanobacteria, could facilitate nutrient recycling and enhance survival in this oligotrophic environment (De los Ríos et al. 2004, 2007). Endolithic communities, dominated by cyanobacteria and containing a diverse assemblage of heterotrophic bacteria closely associated with them within an organomineral matrix, have been previously detected by SEM in samples collected from various desert regions (De los Ríos et al. 2014; Wierzchos et al. 2012, 2015), suggesting shared structural strategies to persist in these extreme environments.

## 8 Potential Mechanisms for Desiccation Tolerance

Using the metagenomic approach, we retrieved several complete microbial genomes or metagenomic‐assembled genomes known as MAGs (Nir et al. 2023; Barak et al. 2023). In our study, the Bin2 MAG (Table S6) was identified at the genus level as *Chroococcidiopsis* in a completion of 99.03% and contamination of 0.44%. Notably, this meets the standards for a high‐quality draft genome, which requires over 90% completeness and less than 5% contamination (Ramos‐Barbero et al. 2019; Kees et al. 2022). Bioinformatic analysis further linked *Chroococcidiopsis* to metabolic capabilities. Here, we highlighted the genes and pathways involved in desiccation tolerance (Table 2). The accumulation of compatible solutes is a key adaptive mechanism in microbial desiccation tolerance (Gasulla et al. 2019; Garcia‐Pichel and Sala 2022). Our study revealed the sugA and sugB genes, which regulate trehalose metabolism, and the susA gene, which regulates sucrose production. The biosynthesis of these solutes may help maintain osmotic equilibrium and support protein function (Lebre et al. 2017; Bosch et al. 2021). Genes encoding heat shock proteins (HSP) were also detected in the *Chroococcidiopsis* metagenome‐assembled genome. These proteins, regulated by thermal stress, appear to play a role in desiccation tolerance (Potts 2001; Tanaka et al. 2004). Acting as molecular chaperones, HSPs perform essential functions such as protein folding and assembly of other proteins. Our study also identified genes encoding chaperone proteins such as clpB, dnaK, DNAj, and surA. These genes have been previously found in cyanobacteria, such as *Anabaena* sp. 7120 (Katoh et al. 2004) and *Ttichocoleus desertorum* isolated from the Negev desert rocks (Nir et al. 2023) where they play a crucial role in the protection and repair of damaged molecules and cell structures in bacterial cells. Several mechanisms have been suggested in previous studies to mitigate oxidative damage resulting from desiccation stress. These mechanisms include the biosynthesis of chemical compounds such as superoxide dismutase (SOD), thioredoxin, and glutamate, and pigments such as carotenoids (Billi 2009; Steimbrüch et al. 2022; Siziya et al. 2022). Our study identified the genes involved in the biosynthesis and regulation of these compounds (Table 2). Finally, our findings point to genes that may be involved in the regulation of DNA repair mechanisms. As an example, it has been shown that the cell damage repair process enhanced the recovery rate of *Chroococcidiopsis* sp. ASB‐02 after desiccation (Billi 2009; Li et al. 2022). Other cellular changes due to xeric stress may include an increase in sheath biosynthesis and thickness. In a previous study on *Chroococcidiopsis* desiccated for up to 66 months, Grilli Caiola et al. (1996) described increased sheath thickness. In *Chroococcidiopsis* sp. ASB‐02, a Mars‐like near‐space environment activated the expression of genes involved in extracellular polysaccharides (EPS). The multilayered cell envelope of the few surviving *Chroococcidiopsis* cells showed a remarkable similarity to the multilayered envelope of *Nostoc* akinetes (Grilli Caiola et al. 1996). Interestingly, *Chroococcidiopsis* maintains a minimal level of monogalactosyldiacylglycerol, which may be essential for the recovery process (Douchi et al. 2024).

To conclude, we collected geological, microbiological, long‐term rainfall, and detailed microclimate records over a three‐year period. Based on these data, we draw the following conclusions.

1. Timna Park is one of the driest locations in the Arava Valley and Israel.
2. The endolithic microbial community in the Timna sandstone was dominated by cyanobacteria, which is consistent with previous work (Friedmann 1980).
3. In the pores of the studied sandstones, both dead and living cyanobacteria coexist along with heterotrophic bacteria that are particularly associated with dead cyanobacteria cells.
4. Lichens or free‐living fungi were absent from both the surface and endolithic habitat in the sandstones of the studied Timna area.
5. Rainfall, although sporadic and light, is the main source of water for endolithic cyanobacteria.
6. Decade‐long droughts occur when the mean annual rainfall is less than 1/3 of the long‐term mean.
7. The described microbial community can survive for extended periods (25 years) under dark, dry conditions without loss of pigmentation or significant changes in community structure.
8. The endolithic cyanobacterial‐dominated community harbours various cyanobacterial taxa that exhibit distinct morphotypes, with the unicellular *Chroococcidiopsis* being predominant.
9. Bioinformatics analysis facilitated the binning of a high‐quality metagenome‐assembled genome (MAG) discovered as *Chroococcidiopsis*, which encompasses multiple genes regulating desiccation tolerance mechanisms.
10. The Timna endolithic community can be a model system for studying the expanded pulse‐reserve paradigm (EPRP) applied to microbial ecosystems in arid environments (Kut and Garcia‐Pichel 2024).

## Author Contributions

**Irit Nir:** conceptualization, writing – original draft, investigation, visualization, data curation. **Rachel Armoza‐Zvuloni:** investigation, methodology, writing – review and editing, data curation, resources, funding acquisition, writing – original draft, visualization, validation. **Hana Barak:** investigation, methodology, data curation, writing – original draft, visualization, validation. **Asunción De los Ríos:** investigation, writing – review and editing, resources, methodology, data curation, writing – original draft, visualization. **Christopher P. McKay:** conceptualization, investigation, writing – review and editing, methodology, resources, data curation, supervision, funding acquisition, writing – original draft, validation. **Ariel Kushmaro:** conceptualization, resources, supervision, methodology, writing – review and editing, funding acquisition.

## Conflicts of Interest

The authors declare no conflicts of interest.

## Supporting information


**Table S1:** emi470188‐sup‐0001‐TableS1.

**Table S2:** emi470188‐sup‐0002‐TableS2.

**Table S3:** emi470188‐sup‐0003‐TableS3.

**Table S4:** emi470188‐sup‐0004‐TableS4.

**Table S5:** emi470188‐sup‐0005‐TableS5.

**Table S6:** emi470188‐sup‐0006‐TableS6.

**Table S7:** emi470188‐sup‐0007‐TableS7.

**Table S8:** emi470188‐sup‐0008‐TableS8.

**Table S9:** emi470188‐sup‐0009‐TableS9.

**Table S10:** emi470188‐sup‐0010‐TableS10.

**Table S11:** emi470188‐sup‐0011‐TableS11.

## Data Availability

The data that supports the findings of this study are available in the Supporting Information of this article.
