# Supplementary material for: The Biology, Microclimate, and Geology of a Distinctive Ecosystem Within the Sandstone of Hyper‐Arid Timna Valley, Israel
Source: Environ Microbiol Rep. 2025 Sep 15;17(5):e70188. doi: 10.1111/1758-2229.70188 (PMC12434837; doi:10.1111/1758-2229.70188)
Supplement: Supplementary file 4 — Table S4: emi470188‐sup‐0004‐TableS4. [file EMI4-17-e70188-s005.docx]

Table S4: Grain size distribution of sandstone rock sample, Timna, Israel

| Aperture (micrones) | Class weight (g) |
| --- | --- |
| 355 | 0.106 |
| 300 | 0.193 |
| 250 | 0.874 |
| 212 | 0.957 |
| 180 | 0.811 |
| 150 | 0.345 |
| 125 | 0.213 |
| 106 | 0.3 |
| 63 | 0.123 |
