# Supplementary material for: The Biology, Microclimate, and Geology of a Distinctive Ecosystem Within the Sandstone of Hyper‐Arid Timna Valley, Israel
Source: Environ Microbiol Rep. 2025 Sep 15;17(5):e70188. doi: 10.1111/1758-2229.70188 (PMC12434837; doi:10.1111/1758-2229.70188)
Supplement: Supplementary file 5 — Table S5: emi470188‐sup‐0005‐TableS5. [file EMI4-17-e70188-s004.docx]

Table S5 supplemetary file

Chemical elements and mineralogical composition of Sandstone samples, Timna Park

Samples from 2021 stones

Element Concentration (%). XRD pattern

1 Si 82.374


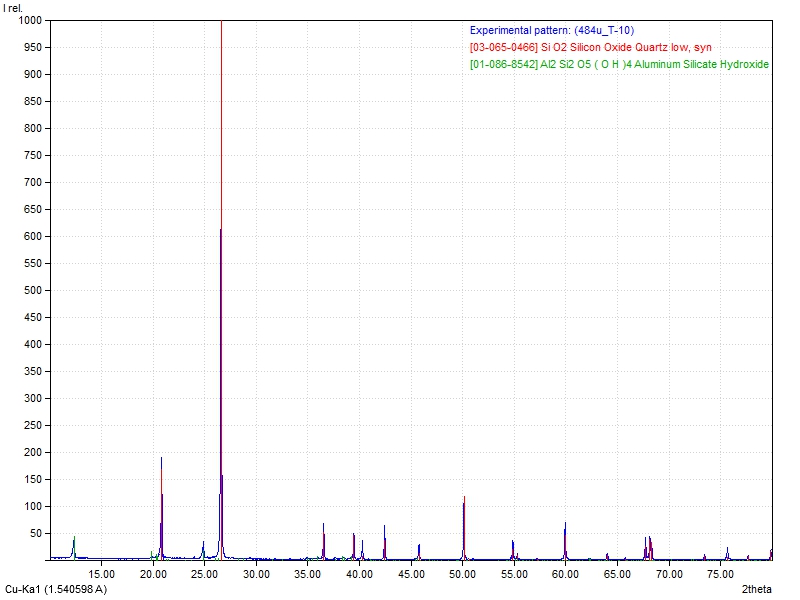
 2 Al 9.064

3 Fe 3.605

4 Ti 1.275

5 Ca 1.229

6 K 0.561

7 Mn 0.438

8 Mg 0.325

9 Cr 0.296

10 Cl 0.230

11 S 0.166

12 P 0.148

13 Zr 0.114

14 Cu 0.062

15 Ni 0.057

16 Pb 0.024

17 Sr 0.019

18 Zn 0.009

19 Y 0.004

Element Concentration (%)

1 Si 87.796 XRD pattern

2 Al 6.986


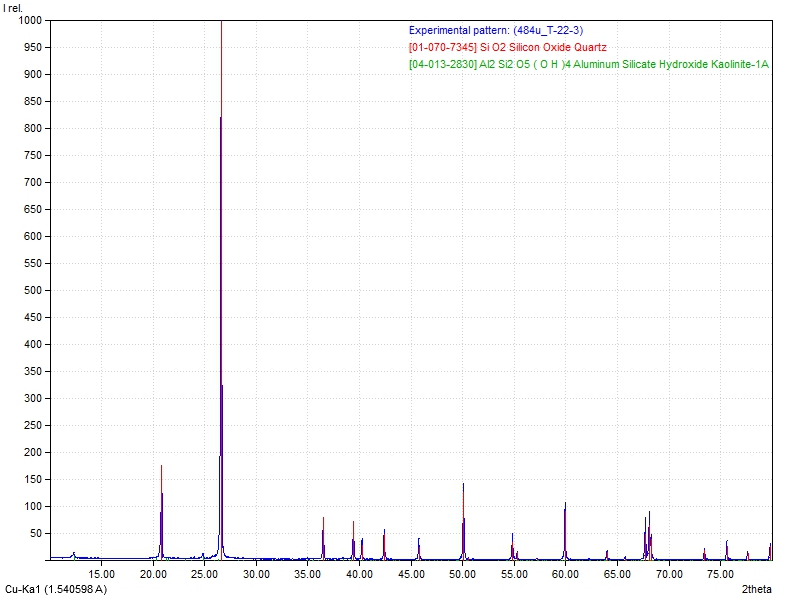
 3 Fe 1.514

4 Ca 0.873

5 Mg 0.513

6 K 0.396

7 S 0.393

8 Mn 0.353

9 Ti 0.292

10 Cl 0.286

11 P 0.268

12 Ba 0.108

13 Sr 0.073

14 Cu 0.053

15 Zr 0.036

16 Ni 0.021

17 Pb 0.019

18 Zn 0.013

19 Y 0.007

Element Concentratiin(%) . XRD pattern


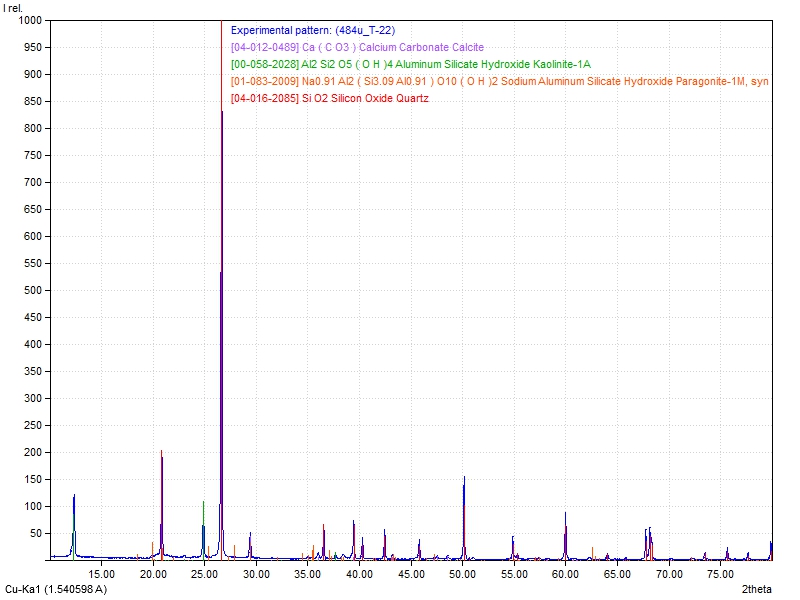
 1 Si 76.188

2 Al 11.968

3 Ca 10.215

4 Ti 0.296

5 Fe 0.252

6 S 0.200

7 K 0.191

8 P 0.176

9 Cl 0.125

10 Cu 0.119

11 Mg 0.117

12 Sr 0.087

13 Zr 0.031

14 Mn 0.020

15 Ni 0.011

16 Y 0.005

Samples from 1995 stone


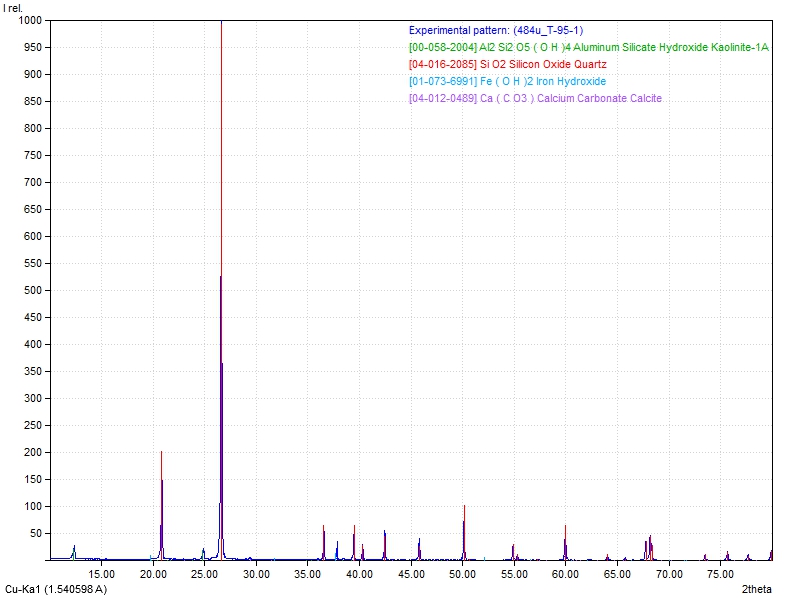
Element Concentration (%) XRD pattern

1 Si 85.821

2 Al 9.367

3 Ca 2.315

4 Ti 0.705

5 Fe 0.648

6 Cl 0.228

7 K 0.227

8 Mg 0.184

9 P 0.156

10 S 0.143

11 Cu 0.084

12 Zr 0.067

13 Sr 0.022

14 Mn 0.019

15 Ni 0.010

16 Y 0.003

**Sample 2**:

Element Concentration (%) XRD pattern


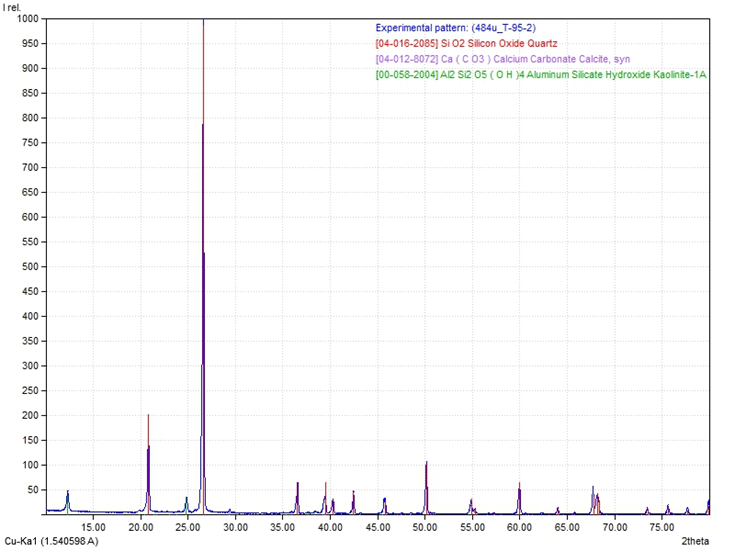
 1 Si 85.553

2 Al 10.270

3 Ca 2.554

4 Ti 0.506

5 Fe 0.328

6 Cl 0.131

7 Mg 0.130

8 K 0.106

9 P 0.098

10 S 0.079

11 Cu 0.077

12 Na 0.057

13 Zr 0.054

14 Cr 0.018

15 Sr 0.017

16 Ni 0.015

17 V 0.008
