# Supplementary material for: The Biology, Microclimate, and Geology of a Distinctive Ecosystem Within the Sandstone of Hyper‐Arid Timna Valley, Israel
Source: Environ Microbiol Rep. 2025 Sep 15;17(5):e70188. doi: 10.1111/1758-2229.70188 (PMC12434837; doi:10.1111/1758-2229.70188)
Supplement: Supplementary file 6 — Table S6: emi470188‐sup‐0006‐TableS6. [file EMI4-17-e70188-s009.docx]

**Supplementary Materials**

**Table S2: Cyanobacteria taxon annotation (Class_Family_Genus)**

| **Taxon (class level)** | **Taxon (family level)** | **Taxon (genus level)** | **T2** | **T6** | **T10** |
| --- | --- | --- | --- | --- | --- |
| Chroococcidiopsidales | Chroococcidiopsidaceae | Aliterella; | 5.42 | 5.20 | 5.33 |
|  |  | Chroococcidiopsis; | 4.91 | 4.91 | 4.82 |
| Nostocales | Chlorogloeopsidaceae | Chlorogloeopsis; | 1.32 | 1.13 | 1.27 |
|  | Hapalosiphonaceae | Fischerella; | 4.11 | 3.97 | 3.91 |
|  | Nostocaceae | Anabaena; | 1.13 | 1.14 | 1.16 |
|  |  | Cylindrospermum; | 1.19 | 1.15 | 1.17 |
|  |  | Nostoc; | 12.87 | 12.50 | 12.40 |
|  | Rivulariaceae | Calothrix; | 6.66 | 6.72 | 6.53 |
|  | Scytonemataceae | Scytonema; | 6.85 | 7.05 | 6.35 |
|  | Symphyonemataceae | Mastigocladopsis; | 1.21 | 1.14 | 1.08 |
|  | Tolypothrichaceae | Tolypothrix; | 3.52 | 3.67 | 3.30 |
| Chroococcales | Chroococcaceae | Chroogloeocystis; | 1.03 | 0.94 | 0.99 |
|  |  | Gloeocapsa; | 1.71 | 1.58 | 1.49 |
|  |  | Gloeocapsopsis; | 3.19 | 2.94 | 3.04 |
|  | Entophysalidaceae | Chlorogloea; | 3.13 | 3.57 | 3.27 |
| Oscillatoriales | Coleofasciculaceae | Coleofasciculus; | 0.94 | 1.01 | 0.93 |
|  | Cyanothecaceae; | Cyanothece; | 1.51 | 1.43 | 1.79 |
|  | Gomontiellaceae; | Crinalium; | 1.53 | 1.51 | 1.54 |
|  | Microcoleaceae | Microcoleus; | 2.31 | 2.26 | 2.21 |
|  | Oscillatoriaceae; | Moorea; | 1.20 | 1.34 | 1.22 |
|  |  | Oscillatoria; | 1.97 | 2.05 | 1.99 |
|  |  | Phormidium; | 1.34 | 1.22 | 1.41 |
| Others | Others | Others | 19.68 | 19.71 | 20.29 |
| Synechococcales | Leptolyngbyaceae | Leptolyngbya; | 5.80 | 6.35 | 6.75 |
|  |  | Phormidesmis; | 1.45 | 1.49 | 1.57 |
|  | Synechocystis; | Synechocystis; | 2.98 | 2.97 | 3.01 |
|  | Synechococcaceae | Synechococcus; | 1.04 | 1.04 | 1.18 |
