# Supplementary material for: The Biology, Microclimate, and Geology of a Distinctive Ecosystem Within the Sandstone of Hyper‐Arid Timna Valley, Israel
Source: Environ Microbiol Rep. 2025 Sep 15;17(5):e70188. doi: 10.1111/1758-2229.70188 (PMC12434837; doi:10.1111/1758-2229.70188)
Supplement: Supplementary file 9 — Table S9: emi470188‐sup‐0009‐TableS9. [file EMI4-17-e70188-s007.docx]

Table S9 supplementary file

Table S9: Stress response genes abundance (%) in Chrooccodiopsis (BIN 2)

| **length_bp** | **gene** | **EC_number** | **COG** | **product** |
| --- | --- | --- | --- | --- |
| **Radiation** | | | | |
|  | uvsE |  | COG4294 | UV DNA damage endonuclease |
|  | uvrA |  | COG0178 | UvrABC system protein A |
|  | uvrB |  |  | UvrABC system protein B |
|  | uvrC |  | COG0322 | UvrABC system protein C |
| Pigments |  |  |  |  |
|  |  | 1.13.11.- | COG3670 | Carotenoid cleavage oxygenase |
|  |  |  |  | Orange carotenoid-binding protein |
| **Heat** | | | | |
|  | clpB1 |  | COG0542 | Chaperone protein |
|  | dnaK2_2 |  |  | Chaperone protein |
|  | clpB |  |  | Chaperone protein |
|  | dnaJ_1-9 |  |  | Chaperone protein |
|  | dnaK_1-3 |  |  | Chaperone protein |
|  | htpG |  | COG0326 | Chaperone protein |
|  | surA_1-2 | 5.2.1.8 |  | Chaperone |
| **Dessication** | | | | |
| EPS |  |  |  |  |
|  | rfaQ | 2.-.-.- | COG0859 | Lipopolysaccharide core heptosyltransferase |
|  | lptC |  |  | Lipopolysaccharide export system protein |
|  | msmX_1-2 | 7.5.2.- | COG3839 | Oligosaccharides import ATP-binding protein |
|  |  | 2.4.1.161 | COG1501 | Oligosaccharide 4-alpha-D-glucosyltransferase |
| Cell wall |  |  |  |  |
|  | ftsI | 3.4.16.4 | COG0768 | Peptidoglycan D,D-transpeptidase |
|  | mrdA | 3.4.16.4 | COG0768 | Peptidoglycan D,D-transpeptidase |
|  | mrdB | 2.4.1.129 | COG0772 | Peptidoglycan glycosyltransferase |
|  | pal |  |  | Peptidoglycan-associated lipoprotein |
|  | patA_1 | 2.3.1.- | COG1696 | Peptidoglycan O-acetyltransferase |
|  | cotA_1 |  | COG2132 | Spore coat protein A |
|  | cotA_2 |  | COG2132 | Spore coat protein A |
|  | hspA |  | COG0071 | Spore protein SP21 |
|  |  | 3.5.1.104 | COG0726 | Peptidoglycan-N-acetylglucosamine deacetylase |
| **Oamotic regulation** | | | | |
|  | sugA |  | COG1175 | Trehalose transport system permease protein |
|  | sugB |  | COG0395 | Trehalose transport system permease protein |
|  | kimA |  | COG0531 | Potassium transporter |
|  | kdpA |  | COG2060 | Potassium-transporting ATPase potassium-binding subunit |
|  | nhaS3 |  |  | High-affinity Na(+)/H(+) antiporter |
|  | nhaP |  |  | K(+)/H(+) antiporter |
|  | nhaP2 |  |  | K(+)/H(+) antiporter |
|  | susA | 2.4.1.13 | COG0438 | Sucrose synthase |
| **Oxidative stress** | | | | |
|  | ggt_2 | 3.4.19.13 | COG0405 | Glutathione hydrolase proenzyme |
|  | gsiA_1_2 | 7.4.2.10 | COG1123 | Glutathione import ATP-binding protein |
|  | kefB_1-3 |  |  | Glutathione-regulated potassium-efflux system protein |
|  | kefC |  |  | Glutathione-regulated potassium-efflux system protein |
|  | sodA2_1 | 1.15.1.1 |  | Superoxide dismutase [Mn] 2 |
|  | sodC | 1.15.1.1 | COG2032 | Superoxide dismutase [Cu-Zn] |
|  | perR |  | COG0735 | Peroxide-responsive repressor |
|  | katA_1 | 1.11.1.6 | COG0753 | Catalase |
|  | trxA_1 |  |  | Thioredoxin 1 |
|  |  |  |  | Thioredoxin-like protein |
|  | trxB |  | COG0526 | Thioredoxin 2 |
| **Other** | | | | |
|  | mprA_1_2 |  | COG0745 | Response regulator |
|  | pleD_1_2 |  |  | Response regulator |
|  | vraR_1 |  |  | Response regulator protein |
|  | yedK | 3.4.-.- | COG2135 | SOS response-associated protein |
|  | sasA_1-29 | 2.7.-.- |  | Adaptive-response sensory-kinase |
|  | yceD |  | COG2310 | General stress protein 16U |
|  | ydaD | 1.-.-.- |  | General stress protein 39 |
| **DNA repair** | | | | |
|  | mutL |  | COG0323 | DNA mismatch repair protein |
|  | recO |  |  | DNA repair protein |
|  | mutL |  | COG0323 | DNA mismatch repair protein |
|  | dps | 1.16.-.- | COG0783 | DNA protection during starvation protein |
|  | dps2 | 1.16.-.- | COG0783 | DNA protection during starvation protein 2 |
|  | radA | 3.6.4.- | COG1066 | DNA repair protein |
|  | recF |  | COG1195 | DNA replication and repair protein RecF |
|  | mutS |  | COG0249 | DNA mismatch repair protein |
|  | recN |  | COG0497 | DNA repair protein |

Stress response genes abundance (%) in Chrooccodiopsis BIN ?
