# Supplementary material for: The Biology, Microclimate, and Geology of a Distinctive Ecosystem Within the Sandstone of Hyper‐Arid Timna Valley, Israel
Source: Environ Microbiol Rep. 2025 Sep 15;17(5):e70188. doi: 10.1111/1758-2229.70188 (PMC12434837; doi:10.1111/1758-2229.70188)
Supplement: Supplementary file 10 — Table S10: emi470188‐sup‐0010‐TableS10. [file EMI4-17-e70188-s001.docx]

Table S10 supplementary file

Cyanobacterial Clones similarities

| Year | Sample | Similarity |  | Similarity |  |
| --- | --- | --- | --- | --- | --- |
| 2021 | Clone1 | 97.81% | MZ677397 Sahara | 94.4% | JF810071/1 |
| 2021 | Clone 2 | 92.58% | MZ677397 | 96.9% | JF810071/1 |
| 2021 | Clone 3 | 97% | MZ677397 | 94.7% | JF810071/1 |
| 2021 | Clone 4 | 97.26% | DQ914865.2 | 94% | AF279107.4 |
| 2021 | Clone 5 | 93.7% | DQ914865.2 | 96% | AF279107.4 |
| 2021 | Clone 6 | 96% | DQ914865.2 | 95% | AF279107.4 |
