# Supplementary material for: The Biology, Microclimate, and Geology of a Distinctive Ecosystem Within the Sandstone of Hyper‐Arid Timna Valley, Israel
Source: Environ Microbiol Rep. 2025 Sep 15;17(5):e70188. doi: 10.1111/1758-2229.70188 (PMC12434837; doi:10.1111/1758-2229.70188)
Supplement: Supplementary file 11 — Table S11: emi470188‐sup‐0011‐TableS11. [file EMI4-17-e70188-s008.docx]

**Study Plan Diagram**

| **Micro-climatic monitoring** | | | **Physical and mineralogical analyses** | | | **Microbial communities’ characterization** | | |
| --- | --- | --- | --- | --- | --- | --- | --- | --- |
| Location | Time scale | |  | 1995** samples | 2021* samples |  | 1995** samples | 2021* samples |
| Local Hill  N29° 46.225', E 34° 56.683' | 1996-1999 | | Pore size | 1X 3 (Table S2) |  | SEM | 3 | 3 |
| Timna Park | 2015-2023 | | Rock drying | Few samples |  | Molecular Study | 3**: T2,** T3, T4 | 3 **(T10**, **T6**, T9) |
| Arava region | 2015-2023 | | Grain size | 1 | 1 | In bold: samples that passed QC and libraries establishment | | |
| Arava region | 1949-2023  (ISM*** Eilat Station) | 1977-2023 (ISM*** Yotvata Station) | XRD analysis | T2, T3 | T9, T10, T6 | Isolation | All samples | |

*2021 samples were collected aseptically and stored room temperature in the microbiology lab (BGU).

** 1995 samples were collected and delivered to NASA-AIMS laboratories and stored in cotton geological bags in a dark closet.

***Israel Meteorological Service (ISM)
